# Nonlocal Effect of Percolated Particle Networks on Viscoelasticity of Polymer–Filler Nanocomposites: A Mesoscale Simulation Study

**DOI:** 10.1021/acs.macromol.6c00792

**Published:** 2026-06-03

**Authors:** Weikang Xian, Zihan Tang, Amitesh Maiti, Andrew P. Saab, Ying Li

**Affiliations:** † Department of Mechanical Engineering, 5228 University of Wisconsin-Madison, Madison, Wisconsin 53706-1572, United States; ‡ 4578 Lawrence Livermore National Laboratory, Livermore, California 94550, United States

## Abstract

With nanoparticles (NPs) as fillers, polymer nanocomposites (PNCs) usually exhibit enhanced mechanical properties. However, a direct connection between the microscopic structural relaxation and macroscopic mechanical properties of PNCs remains to be established. To investigate the micro-to-macro connection, we develop a mesoscale model, in which the NP-bridging polymer chains are represented by a dynamic bonded interaction between NPs, and the bulk polymer matrix is implicitly modeled by overdamped Langevin dynamics. Extensive equilibrium simulations are performed to quantify the microscopic dynamics of model PNCs. Systematic analyses of modified Rouse dynamics, dynamic structure factor, and relaxation modulus uncover that the microscopic relaxation dynamics of PNCs are significantly decelerated across different length scales because of nonlocal effects of percolated particle networks—a phenomenon that has not been adequately captured in prior simulation studies. We find that NP-volume-fraction and NP-bonding-energy barrier are the two critical variables that affect bulk viscoelasticity the most. The proposed mesoscale model is versatile and provides a powerful framework for studying structure–property relations of different PNCs.

## Graphical abstract

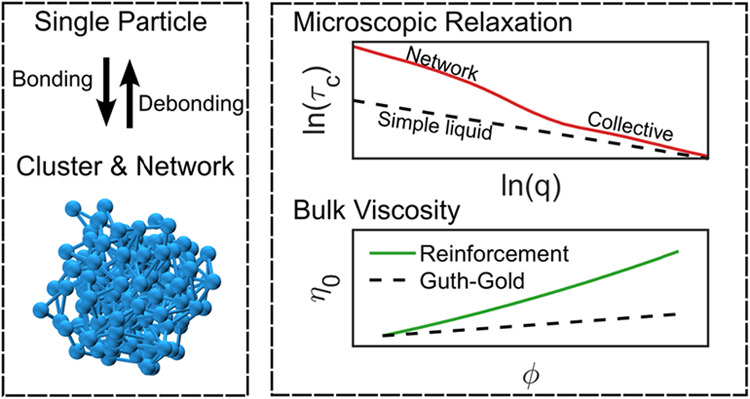

## 1 Introduction

Nanoparticles (NPs) have been used as important fillers to enhance the chemical, thermal, and mechanical properties of matrix polymers. Due to a substantially high surface-to-volume ratio, the strong interactions between the NPs and polymer matrix dominate the properties of the polymer nanocomposites (PNCs). For decades, carbon black NPs have been extensively used in rubber industry for automobile applications because the NPs significantly improve the mechanical stiffness, energy dissipation, antiabrasive, and antiaging properties of the polymer matrices, such as butadiene and styrene–butadiene polymers.^1^ It is well documented that the enhancement of properties originates from the microscopic structures, such as aggregate, agglomerate, and percolated network of the NPs.^2^ Additionally, silica NPs have attracted considerable interest because their surface chemistry can be readily and systematically modified, providing broad opportunities to tailor the inter-NP and polymer-NP interactions.^3^

The hydrodynamic effect was first identified in studies of suspension rheology and attributes the reinforcement to the rigid filler particles disturbing the deformation field of the polymer matrix. The classical Einstein-Smallwood and Guth-Gold models quantify the reinforcement with first- and second-order approximations, assuming the absence of strong interparticle interactions.^4,5^ However, it has been widely reported that the hydrodynamic models fail to adequately quantify the nonlinear reinforcement observed in PNCs, as the NPs strongly interact with the polymer chains in the matrix and may also directly contact one another.^6^ If the interparticle distance *r* is comparable to their effective size *d*, the NPs interact with each other through the polymer chains adsorbed on the particle surfaces, i.e., the bound layer.^7^ If *d* < *r* < *R*_ee_ where *R*_ee_ is the end-to-end distance of the matrix polymer, the NPs interact through bridging polymer chains that are partially adsorbed on the particle surface. In such cases, the NPs effectively serve as junctions that physically cross-link the matrix into micro- or macroscopic networks. Additionally, the bridging chains and the (glassy) bound layer can undergo processes of debonding, desorption, and disentanglement, resulting in irreversible and nonlinear phenomena such as the Mullins and the Payne effects.^8,9^

Although a unified model capable of capturing all these phenomena is not yet available, viscoelasticity remains a key aspect for understanding these phenomena. The viscoelastic properties of a pure polymer matrix are correlated with the conformational relaxation of the constituent molecules.^10,11^ For example, the dynamics of linear polymer melts are well-described by the Rouse^12^ or tube-reptation^13^ models, which capture the conformational evolutions of the Kuhn and tube segments, and thus the chains. The viscoelasticity of PNCs can be understood within a similar multiscale framework, with extra length and time scales introduced by the NPs. To elucidate the viscoelasticity of PNCs across multiple scales, extensive experimental efforts have been made via methods^14^ such as nuclear magnetic resonance (NMR), neutron spin echo (NSE), broadband dielectric spectroscopy (BDS), X-ray photon correlation spectroscopy (XPCS), and transmission electron microscopy (TEM), with a special focus on the polymer-NP interface.^15^ At the chain-segment length scale, the polymer desorption at the NP-interface is a complex, cooperative process accompanied by segmental relaxation that is greatly different from the relaxation of isotropic melts.^6,16–18^ The altered segmental relaxation is dependent on the molecular weight,^19^ level of chain stretching,^20^ and affinity between the NPs and matrix.^21^ In the BDS characterization by Popov et al., it is shown that the magnitude of dielectric response decreases regardless of the chain conformations and relaxation rates of the interfacial chains.^22^ At the polymer-NP interface, the center-of-mass diffusion can be strongly affected by the size, shape, and surface modification of the NPs.^23^ Additionally, the NPs can relax via diffusion or hopping mechanisms, in sharp contrast with the relaxation of polymer chains at the interface or in the matrix.^24–27^

Molecular simulations are powerful tools for investigating the viscoelasticity of PNCs, not only as a supplement to experimental characterizations but also as an approach that stands on its own.^28^ For example, based on generic models, early investigations focused on sampling the static conformations of the interfacial polymer chains.^29–31^ With the growth in computational power, the estimation of viscoelastic responses in the bulk via equilibrium and nonequilibrium simulations was made possible.^32–34^ Recently, all-atomistic molecular dynamics (AAMD) simulations have been utilized to investigate the chemistry-specific structural and dynamic properties of PNCs with silica^35,36^ and carbon-based^37–39^ NPs, confirming the dominating roles of shape and size of the NPs. Aiming to overcome the limitation of length and time scales and to link the microscopic relaxation to the bulk mechanical properties,^40–45^ coarse-grained molecular dynamics (CGMD) simulations have been widely used to quantify the relaxation dynamics and mechanical reinforcement of unentangled and entangled polymer chains in the presence of NPs. Very recently, Mohottalalage et al. used CGMD simulations with up to 10 million beads to show that NPs can form clusters entropically even under weak, nonspecific interactions.^46^ Using a similarly large CGMD model, Kawak et al. showed that the mismatch in the Poisson’s ratios of the NPs and matrix can initiate reinforcement.^47^ AAMD and CGMD simulations are also valuable for understanding the segmental dynamics at the interface of NPs. Using a generic CGMD model to study PNCs with low loadings of NPs, Liu et al. analyzed the incoherent dynamic structure factor (DSF) of the polymer matrix at different distance from the NPs and revealed the deceleration of segmental dynamics.^48^ Li et al. also observed similar deceleration in the simulations of free-standing composite films.^49^ In the cases of medium- and high-loading of NPs, the segmental dynamics is substantially decelerated such that extended relaxation processes are observed in the DSF and Rouse mode analyses.^50^ Although results of the generic CGMD simulations may not be directly compared with experimental measurements, the former can still inform molecular design through complementary theoretical insights.^51–53^ For example, by combining a CGMD model and polymer reference interaction site model (PRISM) theory, Maguire et al. successfully predicted and quantified the static structure and phase behaviors of grafted silica NPs in poly­(styrene-*ran*-acrylonitrile) (SAN) and poly­(methyl methacrylate) (PMMA) matrices, with quantitative agreement with SAXS and TEM characterizations.^54^ CGMD simulations have also been employed to study the diffusion mechanisms of NPs in a polymer matrix. For instance, Kalathi et al. showed substantial deceleration of NPs with a size comparable to the entanglement mesh size of the matrix, while diffusion of smaller NPs is largely determined by the Rouse relaxation of the matrix.^55^

It is well acknowledged that the percolated networks of NPs, either interconnected by rubbery bridging chains or by bound layers, dominate the viscoelasticity and reinforcement of PNCs.^7,56^ Using XPCS to probe the relaxation of silica NPs in the polymer matrix, Yavitt et al. revealed that the collective behavior of the network-forming NPs governs the bulk viscoelasticity, especially the terminal relaxation in the complex modulus.^57^ It is also experimentally shown that the substructures of the percolated networks greatly affect the other mechanical properties of PNCs.^58^ However, the experimental characterizations are inherently limited by their respective accessible spatial and/or temporal scales. On one hand, techniques, such as XPCS, BDS, and TEM, focus on quantifying the microscopic properties of PNCs. On the other hand, mechanical and rheological techniques are more effective in quantifying the macroscopic viscoelasticity of PNCs but often lack a direct connection to the molecular structures and dynamics.^59^

It is important to link the microscopic structural and dynamic properties to the mechanical performance of PNCs, for which the mesoscale models and simulations are particularly valuable. For example, Sotta, Long, and others developed a mesoscale model that captures the glassy state of the bound layer and the morphology of NPs to study the Mullins and the Payne effects of PNCs.^60,61^ Wang et al. used a similar mesoscale model to quantify the linear and nonlinear viscoelasticity of PNCs^62^ and found that the Payne effect can be attributed to the shear-induced alignment of the fractal NPs.^63^ However, a clear connection between the microscopic structural relaxation and the macroscopic mechanical properties has yet to be established. The mesoscale model by Sotta, Long, and others is privately owned and it needs to predefine the percolated particle networks rather than generating the networks according to assigned dynamics.^60,61^ The mesoscale model by Wang et al. does not explicitly include bridging chains and, therefore, is limited in terms of quantifying the percolated particle networks.^62,63^ In particular, the important nonlocal structural and dynamic properties of the percolated particle networks have not been directly linked to the bulk viscoelasticity in these previous studies.

In this work, we develop a mesoscale model to investigate mesoscopic structures that are significantly larger than the individual NPs, without simulating the atomistic details of the NPs and polymer matrix. Importantly, to overcome the shortcomings of prior works, the bridging chains are explicitly modeled as bonding and debonding reactions that are governed by assigned energy barriers, which enable a deterministic investigation of the structure–property relations of the model PNCs. As the volume fraction of NPs approaches and exceeds the percolation threshold, the NPs form network-like structures that significantly affect the viscoelasticity and nonlinear reinforcement of the model PNCs. To quantify the structure–property relation, we propose a modified Rouse model with a time-dependent spring constant that is linked to the diffusion of NPs. Additionally, an analysis of the length-dependent relaxation time serves as a powerful tool for linking structure and property of the PNCs, and more importantly reveal nonlocal effects arising from the percolated particle networks, which has not been reported in previous simulation studies. We describe the model and simulation method in Section 2, while Section 3 details the simulation results with a discussion. We summarize the important findings in Section 4.

## 2 Methods

### 2.1 Mesoscale Model

In the proposed model, a single spherical NP is modeled as an isotropic bead. The NP diameter *d*, mass *m* and thermal energy *k*_B_ *T* set the length, mass, and energy scales, respectively. All physical quantities are expressed in terms of these three basic units. The dynamics is evolved according to the overdamped Langevin dynamics with a friction coefficient ξ and a time step d*t*.^64,65^ All NPs in the simulation box are subject to pairwise interaction described by the Yukawa potential, as in eq 1, which is widely adopted to describe colloidal-like soft interactions^66^

1 $$U_{p}\left(r\right)=U_{0,p}\frac{e^{-\kappa r}}{r},r<r_{c,p}$$

where *r* is the separation distance between the centers of two neighboring NPs, and *r*_*c*,*p*_ is the cutoff distance of the pairwise interaction. The parameters *U*_*p*,0_ and κ are the energy and inverse length, respectively. The particle volume fraction ϕ ranges from 0.002 to 0.2, as listed in Table 1. Additionally, the NPs can be connected by bonds that represent bridging polymer chains, as shown in Figure 1a. A bond is modeled by a multi-Gaussian potential,^67^ as in eq 2

2 $$U_{b}\left(r\right)=-U_{0,b}\ln\left(\sum_{i=1}^{4}\frac{1}{w_{i}\sqrt{\pi /2}}\exp\left(\frac{-2{\left(r-r_{i}\right)}^{2}}{w_{i}^{2}}\right)\right)$$

with four equilibrium distances *r*_*i*_, as shown in Figure 1b. Note that a bond is designed to represent one or many bridging chains that connect different NPs. The parameters of the multi-Gaussian potential are listed in Table 1. Compared with the unit length, the values of *r*_*i*_ imply that the size of bridging chains (e.g., the end-to-end distance) is similar to that of the NPs. Therefore, no entanglement effect is considered here. The choice of such a potential results in local stiffness with the spring constant following *K* ∼ (*r* – *d*)^2^ scaling, as shown in Figure 1c. Compared to a harmonic potential, this choice also enables a more stable simulation with an assigned time step (see below). Additionally, both bonding and debonding are allowed, and they are modeled as chemical reactions. In the bonding reaction, if two NPs are within the cutoff distance, *r*_*c*,*b*_, and do not have any established bond, an attempt to generate a new bond is performed. On the other hand, two NPs that have an established bond are randomly selected to perform a debonding trial. The bonding and debonding reactions are modeled by a Bell model, controlled by the energy barriers *E*_B_ and *E*_DB_, respectively. Each particle can have up to *n*_max,*b*_ = 5 bonds. In each system, the debonding barriers *E*_DB_ are different for the four equilibrium distances, as shown in Figure 1d. Such dependence is motivated by the activation-like interaction between NPs, confirmed by experimental observations.^18^ On the other hand, a constant bonding barrier *E*_B_ is assigned to each system. Systems with four different values of *E*_B_ are studied.

**Figure 1:**
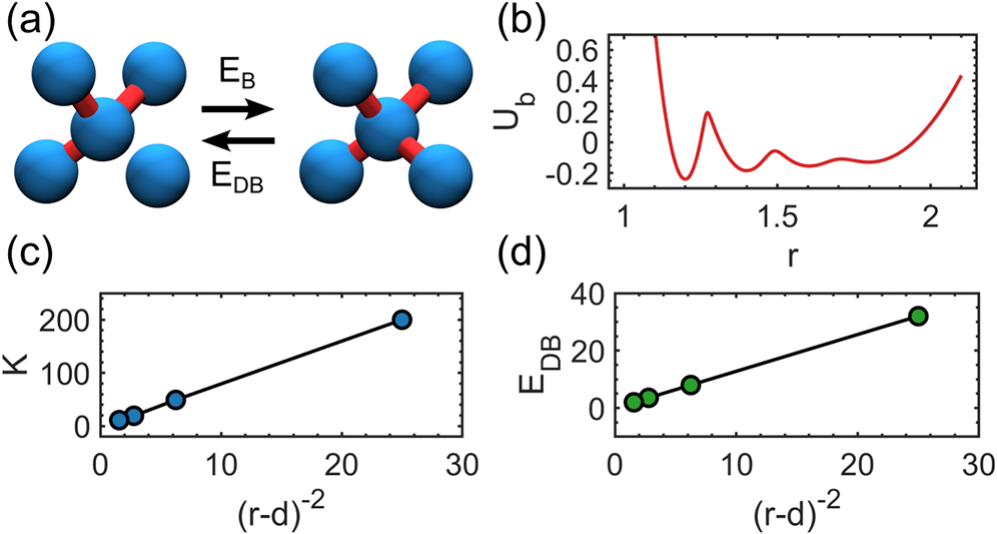
Proposed mesoscale model for PNCs. (a) Individual NPs in blue are modeled by the spherical beads. Bridging polymer chains are modeled by dynamic bonds. The bonding and debonding reactions are controlled by the energy barriers *E*_B_ and *E*_DB_, respectively. (b) The multi-Gaussian potential is used to model the bonded interaction. (c, d) are the local stiffness *K* and debonding energy barriers *E*_DB_ of a bond, respectively. The markers denote the equilibrium distances, *r*_*i*_, per bond. The scaling of ∼(*r* – *d*)^−2^ is consistent with theoretical prediction and experimental observation.^18^ *r* and *d* are the center-to-center distance and diameter of the NPs, respectively.

**Table 1:** Parameters of the Mesoscale Model for the Polymer Nanocomposites

ϕ						
0.002, 0.005, 0.010, 0.015, 0.020, 0.050, 0.075, 0.100, 0.125,0.150, 0.175, 0.200						
*U*_0,*p*_	κ	*r*_*c*,*p*_	*U*_0,*b*_	*w*_1_	*r*_1_	*w*_2_
180.0	5.0	1.1	0.08	0.04	1.2	0.08
*r*_2_	*w*_3_	*r*_3_	*w*_4_	*r*_4_	*r*_*c*,*b*_	*n*_max, *b*_
1.4	0.12	1.6	0.16	1.8	2.1	5
*E*_B_		*E*_DB_(*r*_1_)	*E*_DB_(*r*_2_)	*E*_DB_(*r*_3_)	*E*_DB_(*r*_4_)	*l*_0_
1.5, 2.0, 2.5, 3.0, 3.5		32.0	8.0	3.56	2.0	0.005
τ_MC_	*p*_MC_	*d*	*m*	*k*_B_ *T*	ξ	*dt*
5.0	0.1	1.0	1.0	1.0	100.0	0.001

The Bell model is implemented in a Metropolis Monte Carlo (MC) algorithm.^68^ Specifically, in an MC attempt, a random number 0 < ζ < 1 is generated. A bond is generated if: (1) *r* < *r*_*c*,*b*_ and (2) ζ < e^–*E*_B_^. The pairwise interaction between the two bonded NPs is turned off if a bond is generated. Additionally, an existing bond is annihilated if ζ < e^–(*E*_DB_ *–fl*_0_)^, where *f* and *l*_0_ are the force between two bonded NPs according to eq 2 and the coupling length, respectively. Otherwise, the topology remains unchanged. The MC algorithm is performed every τ_MC_ time-intervals and accepted according to another random process with a preassigned probability *p*_MC_. A list of model parameters is provided in Table 1. Due to the soft-repulsive pairwise interaction and the bonding/debonding reactions, the proposed model setting corresponds to physical systems where the interaction between the NP and polymer is stronger than the interaction between the NPs. The matrix is assumed to be fully compatible with the NPs since no explicit matrix chains are modeled.

### 2.2 Dynamics Simulation

The simulations are conducted using the LAMMPS package developed by SANDIA National Laboratory (version 22-Jul-2025).^69^ The Yukawa pairwise and multi-Gaussian potentials, and Langevin dynamics implemented in the LAMMPS package are used. The bonding and debonding reactions are performed by the REACTION module.^70–72^ For each initial configuration, *N*_total_ = 4 × 10^4^ NPs are randomly placed in a cubic box whose size is given according to the specific volume fraction ϕ. Periodic boundary conditions are applied in all directions. All simulations begin with energy minimization and equilibration for 1 × 10^7^ timesteps without invoking any bonding and debonding reactions. In the second stage of equilibration, the bonding and debonding reactions are turned on and the simulation run for 1 × 10^8^ timesteps, which was found sufficient to ensure that the reactions saturate and the number of bonds remains statistically unchanged. Finally, the production run is carried out for 3 × 10^8^ timesteps. The extensive simulation time ensures statistical convergence for all physical observables. Since there are 5 and 12 different values for *E*_B_ and ϕ respectively, a total of 60 systems are simulated. To track the dynamics of the NPs, the mean-squared displacement (MSD) is calculated as in eq 3

3 $$g\left(t\right)=\left\langle {\left[R\left(t+\tau \right)-R\left(\tau \right)\right]}^{2}\right\rangle$$

where ***R*** is the coordinate of a NP. The ensemble average is performed for all possible combinations of lag (*t*) and reference (τ) times. The diffusion coefficient *D* is evaluated according to $D=\lim_{t\to \infty }g/6t$ . While the MSD reveals the motions of individual particles, it is worthwhile to probe the relaxation behaviors at different length scales. Therefore, the dynamic structure factor (DSF), or often called intermediate scattering function, is calculated as in eq 4

4 $$S\left(q,t\right)=\frac{1}{N_{\mathrm{total}}}\sum_{j,k}^{N_{\mathrm{total}}}\left\langle \exp\left\{iq\cdot \left[R_{j}\left(t+\tau \right)-R_{k}\left(\tau \right)\right]\right\}\right\rangle$$

where ***q*** is the reciprocal wave vector. In isotropic cases, like all systems in this work, it is sufficient to quantify only the magnitude-dependence, such that *S*(*q*,*t*) = *S*(***q***,*t*) is assumed. For each *q* value, 2 × 10^3^ directional vectors that are sampled evenly in the three-dimensional space are used to calculate *S*(*q*,*t*), with *q* ranging from *q*_max_ = 12.5 to *q*_min_ = 4π/*L*(ϕ), where *L*(ϕ) is the ϕ-dependent box size. Additionally, the relaxation modulus *G*(*t*), which quantifies the bulk viscoelasticity, is calculated by the Green–Kubo method as in eq 5

5 $$G\left(t\right)=\frac{V\left(\phi \right)}{k_{B}T}\left\langle \sigma_{\mathrm{ij}}\left(t+\tau \right)\sigma_{\mathrm{ij}}\left(\tau \right)\right\rangle ,i\neq j$$

where σ_*ij*_ is the off-diagonal element of the virial stress tensor without the kinetic energy contribution, and *V*(ϕ) is the ϕ-dependent volume of the model system. *G*(*t*) is calculated on-the-fly by the multitau algorithm,^73^ while *g*(*t*) and *S*(*q*,*t*) are evaluated with our in-house Fortran code. Please refer to a recent discussion by Chen et al. for the *S*(*q*,*t*) calculation.^74^

### 2.3 Modified Rouse Model

A modified Rouse model is presented to further quantify the relaxation of the NPs. The classical Rouse model analyzes the relaxation of a molecule with permanent topology, e.g., a linear polymer chain.^12^ The modified Rouse model, however, explicitly considers the effect of finite topological lifetime on the molecular relaxation. A generalized description is given in eq 6

6 $$dR=-\frac{k\left(t\right)}{\xi }\mathrm{ZR}dt+\sqrt{2D_{0}}dW\left(t\right)$$

where *k*(*t*) is the elastic stiffness of a bond, and variables in bold represent vectors or matrices. ***Z*** is the Rouse-Zimm matrix that fully describes the topology of bonded NPs. Hereafter, a group of bonded NPs is termed as a cluster. *D*_0_ is the diffusivity of a free NP as given by *D*_0_ = *k*_B_ *T*/ξ. ***W*** (*t*) are the vectorized Wiener processes. The coordinates ***R*** (coupled via ***Z***) in Cartesian space can be linearly mapped into a modal space, where the coordinates ***X*** are orthonormal to each other. As a result, the dynamics of each mode are decoupled from each other, as in eq 7

7 $$dX=-\frac{k\left(t\right)}{\xi }\Lambda Xdt+\sqrt{2D_{0}}dW_{p}\left(t\right)$$

In eq 7, **Λ** is the diagonal eigenvalue-matrix of ***Z*** defined by ***Z*** = ***V*** **Λ** ***V*** ^T^, where ***V*** is an orthogonal matrix that connects coordinate systems ***R*** and ***X*** through the definition ***R*** = ***VX***, and the columns of ***V*** represent the orthonormal eigenvectors of ***Z***. **Λ** has diagonal elements λ_*p*_ that correspond to the normal modes *p*. The MSD of a cluster is related to the expectation of all the modes as in eq 8

8 $$g_{i}\left(t\right)=\frac{1}{N_{i}}E\left[\sum_{p=0}^{N_{i}-1}{\left(X_{p}\left(t\right)-X_{p}\left(0\right)\right)}^{2}\right]$$

where *E*[■] represents the expectation operation, and *N*_*i*_ is the number of NPs in the specific cluster *i*. The bulk MSD is calculated as the number-average of the individual contributions, according to *g*(*t*) = ∑θ_*i*_ *g*_*i*_(*t*) where θ_*i*_ = *N*_*i*_/*N*_total_ is the proportion of clusters that are of size *i*. In the case of a permanent topology and constant bond stiffness, i.e., *k*(*t*) = *k*_0_ and *a*_0_ = λ_*p*_ *k*_0_/ξ, the expectation is given in eq 9

9 $$E\left[{\left(X_{p}\left(t\right)-X_{p}\left(0\right)\right)}^{2}\right]={\left(e^{-a_{0}t}-1\right)}^{2}X_{p}^{2}\left(0\right)+\frac{D_{0}}{a_{0}}\left(1-e^{-2a_{0}t}\right)$$

Note that *X*_*p*_ is an individual degree-of-freedom but ***X***_***p***_ is a vector. If the elastic springs follow an exponential decay, i.e., *k*(*t*) = *k*_0_ e^–*t*/τ_*k*_^, and *a*(*t*) = *a*_0_ e^–*t*/τ_*k*_^, the expectation is given in eq 10

10 $$E\left[{\left(X_{p}\left(t\right)-X_{p}\left(0\right)\right)}^{2}\right]={\left(e^{-a_{0}t}-1\right)}^{2}X_{p}^{2}\left(0\right)+2D_{0}B^{2}\left(t\right)I\left(t\right)$$

where *B*(*t*) = exp [−*a*_0_τ_*k*_(1 – e^–*t*/τ_*k*_^)] and *I*(*t*) = τ_*k*_ **e^2*a*_0_τ_*k*_^[*E*_1_(2*a*_0_τ_*k*_ **e^–*t*/τ_*k*_^) – *E*_1_(2*a*_0_τ_*k*_)]. Please refer to the Supporting Information for detailed derivation of the above equations. Due to the presence of the $E_{1}\left(x\right)=\int_{x}^{\infty }\frac{e^{-t}}{t}dt$ integral in eq 10, the modified Rouse model reveals nontrivial relaxation of the clusters of NPs.

## 3 Results and Discussion

### 3.1 Static Structural Properties

Selective snapshots of model systems are shown in Figure 2. In the low-ϕ cases, a great number of small clusters are formed, as shown in Figure 2a. However, a great portion of the NPs are not connected to any cluster (not shown). Please refer to the panels for other selective cases as shown in Figure S1. In the medium-ϕ cases, it is interesting that infinitely large networks are formed across the periodic boundaries, while many other smaller clusters coexist, as shown in Figure 2b.

**Figure 2:**
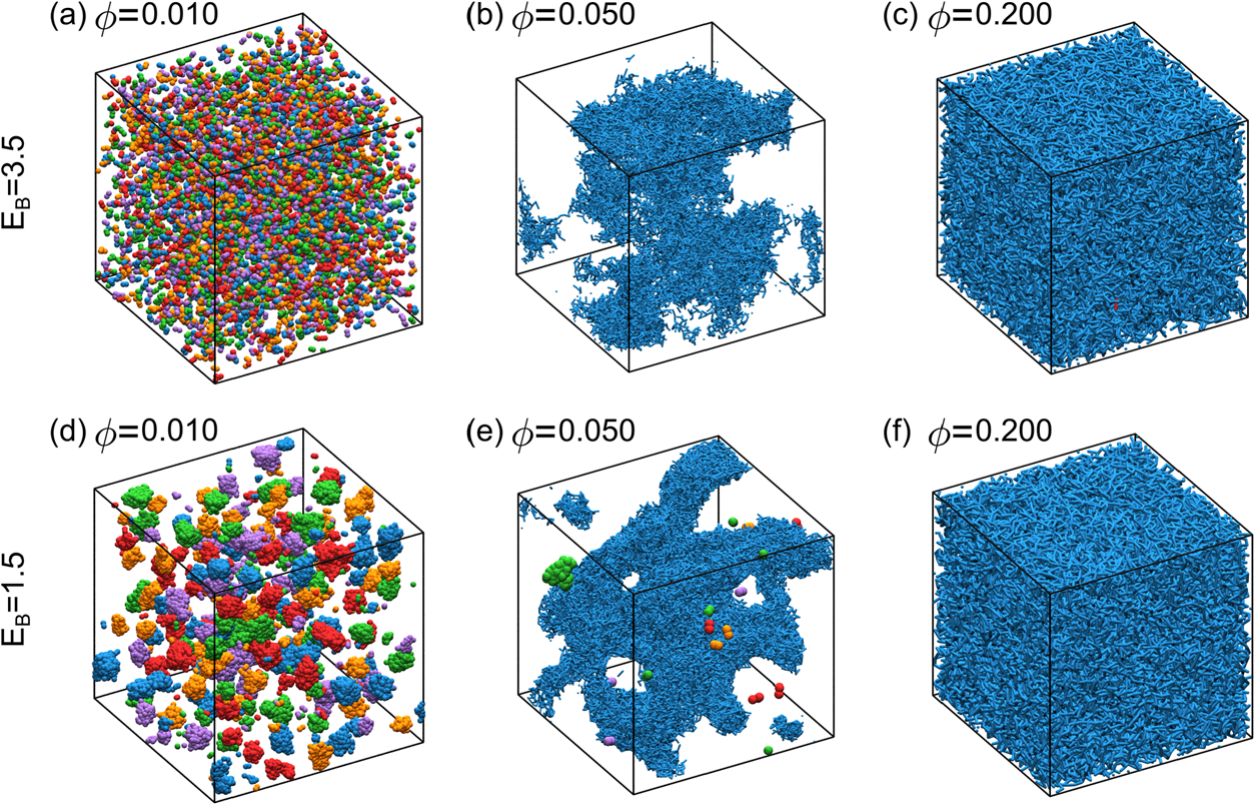
Snapshots of the simulations. (a–c) are for the systems with *E*_B_ = 3.5. (d) to (f) are for the systems with *E*_B_ = 1.5. The volume fraction ϕ = 0.050 is identified as the percolation threshold as it is associated with the emergence of the infinitely large networks (in blue). Below the threshold, the NPs remain freestanding or form clusters. Approaching the threshold, individual NPs, clusters and infinitely large networks coexist, as shown in (e). Above the threshold, the percolated networks include almost all NPs.

Specially, ϕ = 0.050 is identified as the percolation threshold because of the coexistence of many individual clusters and an infinitely large NP network across the periodic boundaries. Interestingly, the above identified percolation threshold agrees well with experimental observations of ϕ = 0.022 to 0.07 for systems in which hydrophobic silica filler particles with diameter *d* ≈ 10 nm are incorporated into a polymer matrix in rubbery state,^75,76^ suggesting that the proposed mesoscale model effectively captures the network-like behavior of PNCs. In the high-ϕ cases, as shown in Figure 2c, almost all NPs interconnect to each other and form a tight network through the periodic boundaries. Additionally, the ϕ-dependent trend is also observed in other cases with different *E*_B_, e.g., *E*_B_ = 1.5, as shown in Figure 2d–2f, for the low-, medium-, and high-ϕ cases, respectively. However, in contrast to the *E*_B_ = 3.5 cases, the individual clusters in the *E*_B_ = 1.5 system are much bigger. With ϕ = 0.050, the supramolecular network in the *E*_B_ = 1.5 system includes more NPs with a stronger local heterogeneity compared with the counterpart in the *E*_B_ = 3.5 system. The “tight” networks in the high-ϕ cases share similar characteristics and are independent of *E*_B_. Lastly, it is worth noting that a high value of *E*_B_ results in a “loose” network, due to the related difficulty in the bonding reaction, while a small values of *E*_B_ facilitates the formation of a “tight” network.

In addition to the visualizations shown in Figure 2, the structural characteristics are quantified by the radius-of-gyration *R_g_* of clusters, as a function of the cluster size *N*. Figure 3 shows representative *R*_*g*_-*N* results that correspond to the cases shown in Figure 2. In the cases of low-ϕ (Figure 3a), all clusters are of size *N* < 20 (note that NP is not included here). At the percolation threshold (Figure 3b), the distribution of *R*_*g*_ covers a wider range up to around *N* = 100, and the percolated particle network is represented by a single point with *N* > 2000. Note that according to the *R*_*g*_ ∼ *N* ^1/*d*_*f*_^ scaling, a trend with *d*_*f*_ = 2.0 is clearly displayed, suggesting a noncompact, branch-like nature of the individual clusters, in agreement with experimental observations.^77^ In the high-ϕ cases, due to the existence of the infinitely large networks, an extra treatment is applied to select local network topology to estimate the *R*_*g*_-*N* relation. In the first step, a random NP that belongs to the network is selected as the center probe. In the second step, within a given cutoff (2.0 to 15.0), the other NPs that are directly and indirectly connected to the center probe are selected. Lastly, the *R*_*g*_ value of the selected NPs is calculated. Such a process is repeated 100 times for one simulation frame. In Figure 3c, it is shown that the trend of *d*_*f*_ = 3.0 dominates, confirming the formation of a tightly packed three-dimensional network. Such characteristics are also observed in the *E*_B_ = 1.5 cases. Interestingly, for the low-ϕ case, the *R*_*g*_ distribution shows a bimodal distribution, as shown in Figure 3d. For the medium-ϕ case, the variation in *N* increases for local network topologies with fixed *R*_*g*_. Note that the special treatment is applied to obtain the results shown in Figures 3c,e, and f. All *R*_*g*_-*N* results are shown in Figures S2–S6. It is worth noting that the emerging bidisperse pattern is observed for the systems with low-ϕ regardless of *E*_B_, and is stronger with lower *E*_B_ values, as a result of the stronger bonding reaction. Regarding the identified percolation threshold ϕ = 0.050, it is not exclusively determined by a single model parameter. It is collectively determined by the model parameters, such *n*_max,*b*_, and the soft-repulsive Yukawa potential. Therefore, it requires extra investigation to establish an exact mapping between the model parameters and the percolation threshold, which is beyond the scope of the current work.

**Figure 3:**
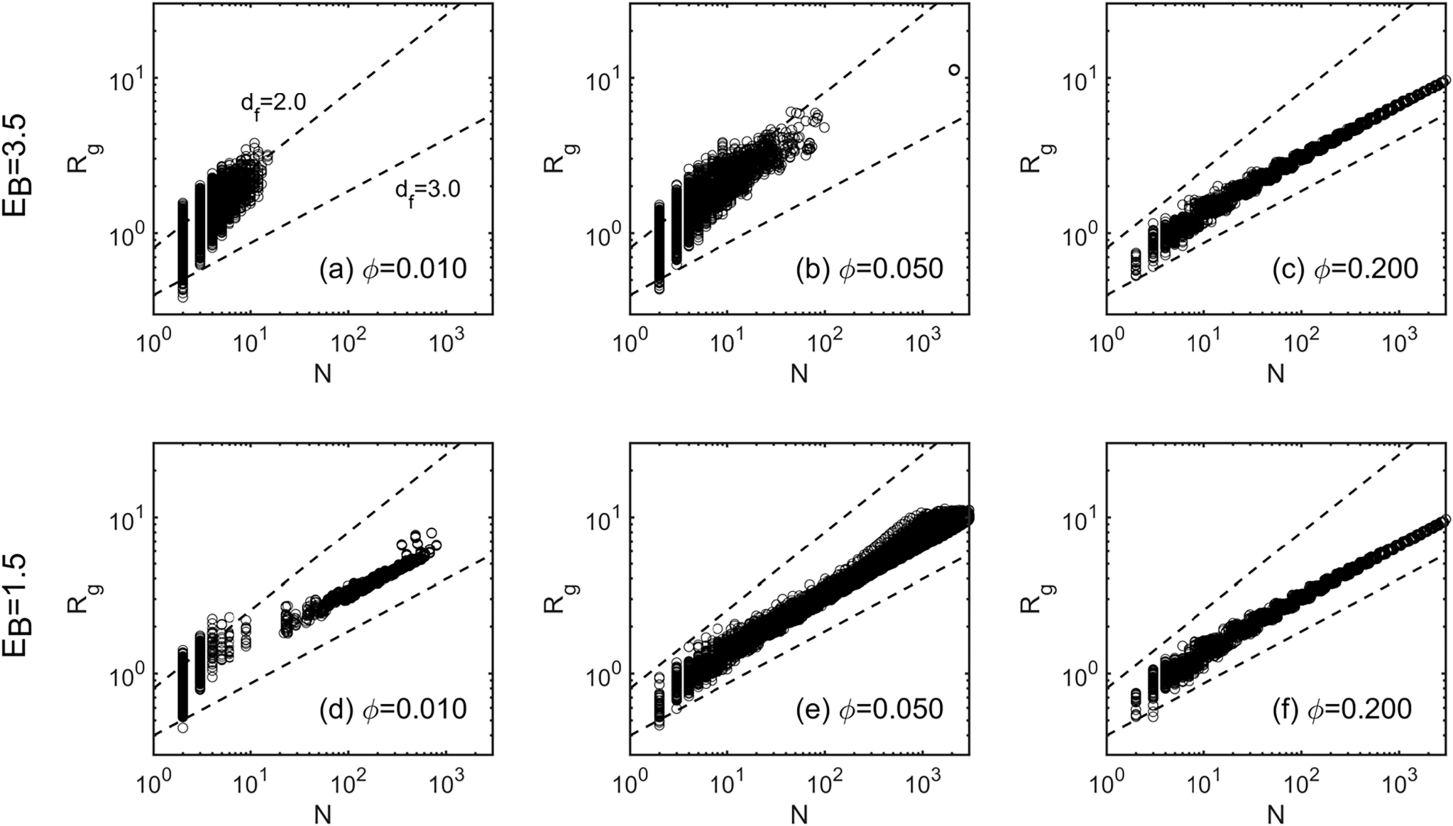
Radius-of-gyration *R*_*g*_ examined as functions of the cluster size *N* via the scaling law *R*_*g*_ ∼ *N* ^1/*d*_*f*_^. (a–c) are for the systems with *E*_B_ = 3.5. (d–f) are for the systems with *E*_B_ = 1.5. Below the percolation threshold, the *R*_*g*_-*N* relation follows a *d*_*f*_ = 2.0 scaling, suggesting noncompact, branch-like structures of the clusters. As *E*_B_ decreases or ϕ increases, large clusters and networks emerge and the *R*_*g*_-*N* relation follows a *d*_*f*_ = 3.0 scaling, suggesting tightly packed structures.

Additionally, the static structure factors at different conditions are calculated, and the results are shown in Figure 4. For the systems with *E*_B_ = 3.5, the *S*_*q*_ curves are relatively flat, reflecting the large number of small clusters, as long as ϕ is below the percolation threshold. However, the *S*_*q*_ curves in the low-*q* regime show significant increase as ϕ reaches the percolation threshold, as shown in Figure 4a. Specifically, the fact that *S*_*q*_ ∼ *q* ^–3.0^ in the low-q regime suggests densely packed morphology at large length scales, which is consistent with previous theoretical predictions and experimental measurements.^78^ Such an observation is clearer in the cases of *E*_B_ = 1.5, as shown in Figure 4b.

**Figure 4:**
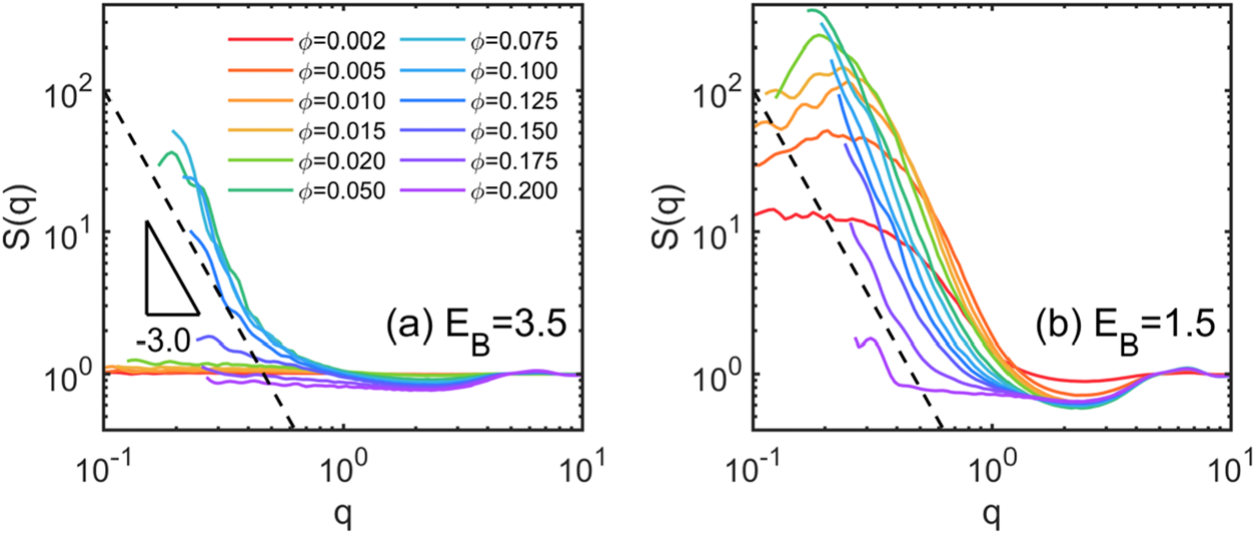
Static structure factor. (a) In the case of *E*_B_ = 3.5, the systems with ϕ = 0.05 to ϕ = 0.125 show a *S*_*q*_ ∼ *q* ^–3^ scaling in the low-*q* regime, while the other systems are in a more homogeneously packed state. (b) In the case of *E*_B_ = 1.5, almost all systems show the *S*_*q*_ ∼ *q* ^–3^ pattern in the low-*q* regime. Moreover, the fluctuations at *q* ≈ 3.0 are also stronger than the systems with *E*_B_ = 3.5.

Since the bonding energy barrier is lower at *E*_B_ = 1.5 and the bonding reaction is more frequent, the probability of the NPs forming larger clusters is much higher than in the case of *E*_B_ = 3.5. Therefore, at low *E*_B_ the *S*_*q*_ ∼ *q* ^–3.0^ scaling is also observed even for systems with low-ϕ, confirming the structural characteristics of the visualizations shown in Figure 2. It is worth noting that for both ϕ = 0.2 cases, the *S*_*q*_ curves do not conclusively show the ∼*q* ^–3.0^ scaling, due to the limited size of the simulation boxes. The results of the systems with *E*_B_ = 3.0 to *E*_B_ = 2.0 are shown in Figure S7. These network-like structures result in nonlocal dynamic characteristics that significantly affect the bulk viscoelasticity of model PNCs.

### 3.2 Bond Lifetime

The microscopic evolution of bonding and debonding reactions dictates the complex bulk viscoelasticity of the model polymer nanocomposites. Here we first evaluate the bond lifetime τ_*b*_ to quantify the evolution of individual bonds.

The bond lifetime is explicitly tracked by the probabilities *P*, defined as the time *t* that a bond lasts. The probabilities *P* decay exponentially as shown in Figure 5a, where the case with *E*_B_ = 1.5 and ϕ = 0.2 is shown. It is implied that the debonding reaction is fully activated, and the model system is far away from the glass transition state (i.e., *T* ≫ *T*_g_). A stretched exponential function *P*(*t*) = exp­(−(*t*/τ_b_^*^)^β^) is fitted against the exponential decay of *P* to estimate the effective bond lifetime τ_*b*_ = τ_b_^*^Γ­(1/β)/β, where Γ is the γ function, and τ_b_^*^ and β are fitting parameters. A rough estimation of τ_*b*_ = τ_MC_/(*p*_MC_ exp (−*E*_DB_)) ≈ 370.0 is suggested based on the simulation parameters. *E*_DB_ = 2.0 is used for the estimation. However, the effective bond lifetime τ_*b*_ are much higher than the rough estimation, as shown in Figure 5b. The comparison between the roughly estimated and effective τ_*b*_ suggests that the latter are collectively affected by the topologies of clusters or networks, complexities in the reactions, and the competition between reactions and diffusion, as described below. For most cases, τ_*b*_ ≈ 2000.0 is observed when ϕ ≥ 0.05. In such cases, there are sufficient NPs available for a center NP to bond in the percolated network adjacent to the center NP. Therefore, τ_*b*_ is insensitive to ϕ because the reaction is significantly faster than the displacement of NP. Conclusively, the reaction is diffusion-limited when ϕ ≥ 0.05. However, when ϕ < 0.05, τ_*b*_ significantly increases as ϕ decreases, especially if *E*_B_ is high (e.g., *E*_B_ = 3.5). It is suggested that the NPs favor in bonding to the previously bonded NPs after the bond-breakage, instead of finding new partners to bond. Specifically, the debonding and rebonding are localized within the individual clusters since ϕ is low. The existence of the two τ*_b_* regimes suggests that the relative contributions and mechanisms from the reactions within the clusters and networks. Additionally, the bond-reformation is stronger with higher *E*_B_ because a cluster is more stable with higher *E*_B_. On the contrary, a lower *E*_B_ value facilitates the formation of new bonds that may not be stable.^68^ More importantly, the volume fraction ϕ significantly affects τ_*b*_ because the former controls the morphologies of the clusters and networks of the model PNC. ϕ and *E*_B_ are two independent variables in the simulation, while other model parameters are control variables. Our analyses of the static structural properties and microscopic bond lifetime clearly differentiate the effects of the bonding and debonding reactions (related to *E*_B_ and *E*_DB_) from the morphologies of the clusters and networks (related to ϕ). In particular, the result shown in Figure 5 clearly highlights that increasing τ_*b*_ with decreasing ϕ is attributed to the fact that NPs are more likely to rebond with the older partners after bond breakage, considering the individual clusters are dominant in the low-ϕ cases as shown in the snapshots in Figure 2.

**Figure 5:**
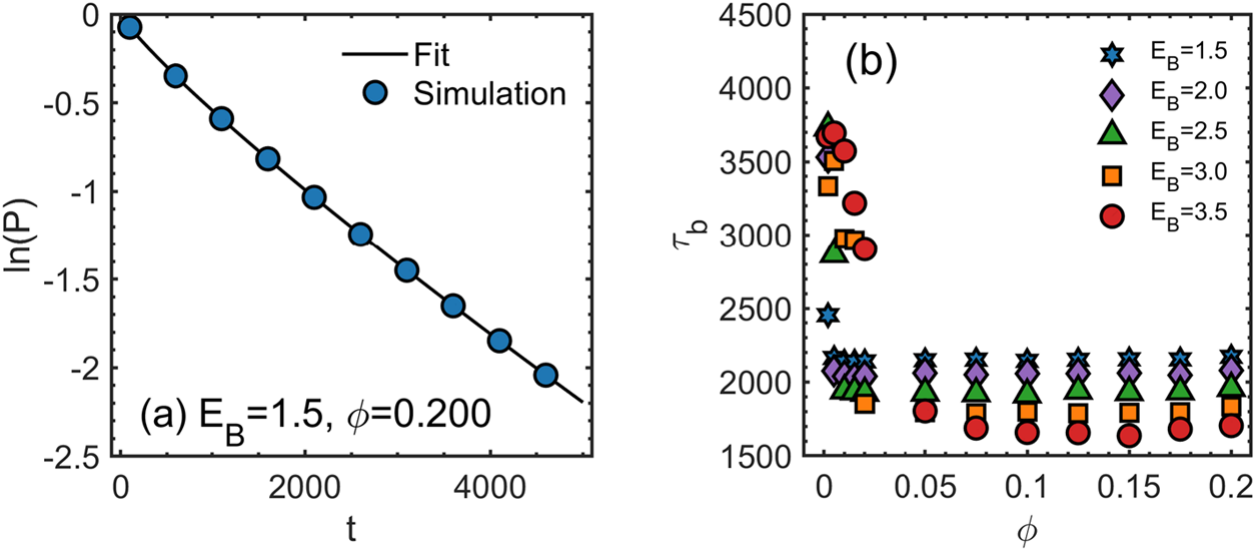
Characteristic of the debonding reaction. (a) Taking the system with *E*_B_ = 1.5 and ϕ = 0.2 as an example, the probability *P* that a bond lasts for time *t* decays. The effective bond lifetime τ_*b*_ is estimated. (b) All τ_*b*_ results are shown as functions of ϕ and *E*_B_. Above the percolation threshold, τ_*b*_ is less sensitive to ϕ. However, τ_*b*_ increases as ϕ decreases. In such cases, a bond tends to rebond with the previous partner after a breakup, such that the τ_*b*_ is effectively increased.

### 3.3 Mean-Squared Displacement

To investigate how the bonding and debonding reactions affect the relaxation of individual NPs, the ensemble-average of MSD results are evaluated. The rescaled *g*(*t*) results of the *E*_B_ = 3.5 systems are shown in Figure 6a as an example. All other MSD results are shown in Figure S8. When ϕ is low (e.g., ϕ = 0.002), *g*(*t*) scales linearly with *t*, suggesting an expected diffusive characteristic. Interestingly, plateaus emerge as ϕ increases, particularly when ϕ is high (e.g., ϕ = 0.2). The scaling *g*(*t*) ∼ *t* ^1/2^ suggests a Rouse-like behavior. In the high-ϕ cases, on one hand, the NPs strongly percolate into supramolecular networks such that the classical Rouse model cannot be applied to quantify the relaxation process of the NPs, since the classical Rouse model only applies to molecular structures with finite degrees-of-freedom. On the other hand, unlike highly constraint systems such as permanently cross-linked networks, the supramolecular networks of NPs are subject to long-term morphological change because of the bonding and debonding reactions. As a result, the bulk mechanical properties of the model PNCs are indeed time-dependent and dictated by the microscopic dynamics of the NPs. The diffusion coefficient is evaluated according to $D=\lim_{t\to \infty }g/6t$ for all systems, and the results are shown in Figure 6b.

**Figure 6:**
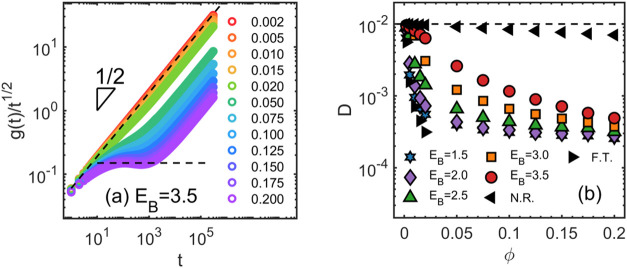
Mean-squared displacement. (a) Taking the systems with *E*_B_ = 3.5 as examples, the magnitude of the MSD decreases as ϕ increases, suggesting a deceleration in the relaxation. At and above the percolation threshold, the ∼*t* ^1/2^ plateaus emerge, indicating Rouse-like relaxation in contrast with the ∼*t* ^1^ behavior of a simple liquid for the systems with low ϕ. (b) Estimated diffusion coefficient *D* as functions of *E*_B_ and ϕ. Note that the results of the fixed-topology (F.T.) and no-reaction (N.R.) systems are also included for a direct comparison. The topologies are permanent in the F.T. systems. The F.T. and N.R. systems do not have any reactions.

Note that *D* includes both contribution from the free NPs and the formed clusters in various sizes. For the sake of comparison, results of two additional types of systems are provided: (1) with the bonding and debonding interactions deactivated completely, labeled as N.R. systems; (2) with the fixed topologies adopted from the corresponding *E*_B_ = 1.5 cases and labeled as F.T. systems. For the N.R. systems, the diffusion coefficient is *D* ≈ *D*_0_ and nearly independent of ϕ. With the bonding and debonding reactions activated, *D* decreases by at least an order of magnitude, comparing the ϕ = 0.002 and ϕ = 0.200 cases. Additionally, the ϕ-dependent nonlinearity is stronger with a lower *E*_B_ since it is easier to form bonds with a lower *E*_B_. In the *E*_B_ = 1.5 cases, the decrease in *D* as a function of ϕ is the most significant, implying the bonding reaction fundamentally decelerates the relaxation of NPs. The *D* asymptotically approaches *D* ≈ 2.0 × 10^–4^ when ϕ ≥ 0.05. Moreover, for the F.T. systems, *D* shows the strongest nonlinearity. Note that no *D* is evaluated for the F.T. systems with ϕ ≥ 0.05 since the networks are infinitely large and permanent, and there is no diffusion at all.

An attempt to predict the diffusion coefficient *D* is also performed. We first focus on the F.T. systems. The estimated diffusion coefficient *D* of systems with ϕ < 0.05 can be well quantified by the classical Rouse model (eq 9) as shown in Figure S9, where the prediction accurately matches the simulation results. In these cases, the topologies and coordinates of individual clusters and NPs are directly used as input. However, percolated networks emerge if ϕ ≥ 0.05, and there is no Fickian diffusion for these percolated systems. Therefore, the classical Rouse model without considering the free diffusion is used to understand the capped MSD curves, which are shown in Figure S10. Specifically, for the capped MSD prediction, the first step is the identification of local network topologies with different cutoff distances *r*_*c*,MSD_. Second, the capped MSD curves are calculated using the classical Rouse model with the zero-eigenvalues (free diffusion) ignored. The two-step operation is repeated for all systems with ϕ ≥ 0.05. By matching the Rouse prediction and the simulation results, the effective cutoff distance *r*_*c*,MSD_ is found ϕ-dependent, and the results are shown in Figure S11. For the systems with active bonding and debonding reactions, the classical Rouse model does not apply because of the time-dependent network. Therefore, the modified Rouse model (eq 10) is used to estimate *D*. For the ϕ < 0.05 systems, a constant τ_*k*_ = 3000.0 is used. The results are shown in Figure S9. The prediction quantitatively matches the simulation result, suggesting that the relaxation of the NPs is indeed controlled by the bonding and debonding reactions.

For the ϕ ≥ 0.05 systems, local network topologies are first identified by the estimated *r*_*c*,MSD_ from the F.T. systems and then used as input for the modified Rouse model to estimate the MSD curves. In Figure 7a, taking the ϕ = 0.2 and *E*_B_ = 3.5 system as an example, the bulk MSD is based on the ensemble-average of all modal contributions from all clusters. A representative modal curve (one single red curve) shows three distinctive regimes, compared with the diffusive limit of *D*_0_ = 0.01: (1) The initial stage with a ∼*t* scaling represents the unrestricted motion of the NPs since the bonded topology is not yet “felt”; (2) The second stage is characterized by the decreased *X*_*p*_(*t*)/6*t* values due to the bonded topology; (3) Since the elastic stiffness (quantified by τ_*k*_) in the modified Rouse model decays exponentially, i.e., *k*(*t*) ∼ exp­(*t*/τ_*k*_), the topological constraint eventually vanishes and thus the NPs restore a diffusive motion, leading to the third stage of relaxation with ∼*t* scaling. It is worth noting that the modified Rouse model degenerates to the classical Rouse model if τ_*k*_ → ∞, and thus there will be only two stages, as shown in Figure S12 by an exemplary MSD of the classical Rouse model. Also note that the third diffusive stage is not observed in the mesoscale dynamics simulation, regardless of ϕ, because the active bonding reactions prevent the vanishment of topological constraint and keep the diffusion bounded. Therefore, the ensemble-average plateau *g*(*t*)/6*t* in the second stage is evaluated as the effective diffusion coefficient *D* in the bulk. Since the estimated *D* is dependent on various as the characteristic time of the elastic stiffness τ_*k*_, different *D*-τ_*k*_ pairs are evaluated, and the effective τ_*k*_ values are interpolated or extrapolated by matching the simulation and modified Rouse prediction results of *D*, as shown in Figure S13. The effective τ_*k*_ are shown in Figure 7b as functions of ϕ and *E*_B_ for all percolated systems. The effective τ_*k*_ respectively increases with ϕ and *E*_B_, suggesting the dominant roles of the two parameters. Interestingly, the results of τ_*k*_ show stronger dependencies on ϕ and *E*_B_ than the results of τ_*b*_ shown in Figure 5b, implying that ϕ and *E*_B_ indeed result in significant changes of bulk material-response although the microscopic bond lifetime τ_*b*_ is less insensitive to ϕ and *E*_B_. The strong dependencies of τ_*k*_ on ϕ and *E*_B_ also imply the significant nonlocal effect due to the percolated particle networks for the ϕ ≥ 0.05 systems.

**Figure 7:**
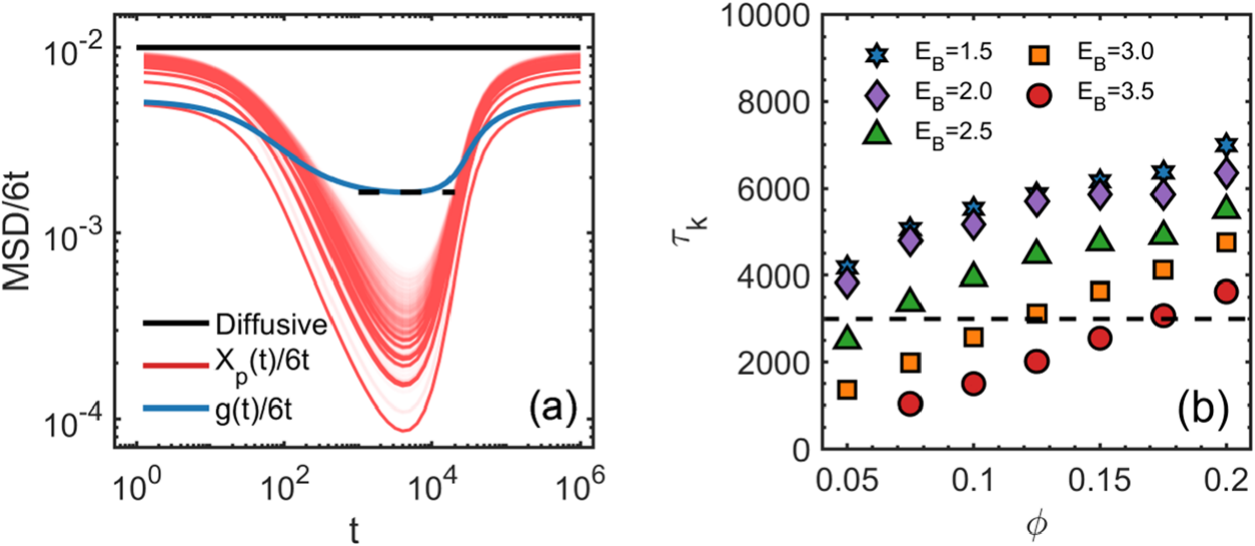
Relaxation dynamics estimated by the modified Rouse model. (a) The contributions of individual modes to the bulk diffusion coefficient, marked by the black dashed line. The results of the system with *E*_B_ = 3.5 and ϕ = 0.02 are used as an example. (b) In the case of percolation, no individual cluster is available for applying the modified Rouse model. The characteristic time τ_*k*_ is estimated instead of directly predicting diffusion coefficients. The τ_*k*_ results suggest the important effects of *E*_B_ and ϕ on the relaxation of the networks.

### 3.4 Dynamic Structure Factor

While the diffusion coefficient *D* reveals the decelerated motions of the NPs subject to the bonding and debonding reactions, an analysis of the *q*-dependent processes provides deeper insight into the relaxation dynamics. For a simple liquid, such as the N.R. systems, the relaxations indicated by the normalized DSF show a uniform *q*-dependence, as shown in Figure S14. Specifically, the DSF exponentially decays with *q*-dependent characteristic time τ_*c*_, i.e., *S*(*q*,*t*)/*S*(*q*,0) = exp­(−*t*/τ_*c*_). The normalized DSF *S*(*q*,*t*)/*S*(*q*,0) results are fitted against the exponential decay, and the τ_*c*_ ∼ *q* ^–2^ scaling is clearly shown in Figure S15.

The τ_*c*_ ∼ *q* ^–2^ scaling is also observed in the case of weak percolation effect, such as in the system with ϕ = 0.01 and *E*_B_ = 3.5, as shown in Figure 8a. However, extended relaxations emerge in the case of stronger bonding and debonding reactions. For example, as shown in Figure 8b, there is an extended tail in the long term decay at the small *q* regime, for the system with ϕ = 0.05. As ϕ increases, the peak shifts toward the regime with higher *q*, as shown in Figure 8c for the system with ϕ = 0.2. In the case of even stronger bonding reaction (e.g., *E*_B_ = 1.5), the extended relaxations emerge even in the low-ϕ cases, such as the ϕ = 0.01 system that is shown in Figure 8d. The cases, with stronger extended relaxation, for ϕ = 0.05 and ϕ = 0.2 are shown in Figure 8e,f, respectively. Compared with the counterparts with *E*_B_ = 3.5, multiple nonuniform peaks are shown in the *E*_B_ = 1.5 cases, suggesting that a higher *E*_B_ results in a stronger percolation effect. All DSF results are shown in Figures S16–S20.

**Figure 8:**
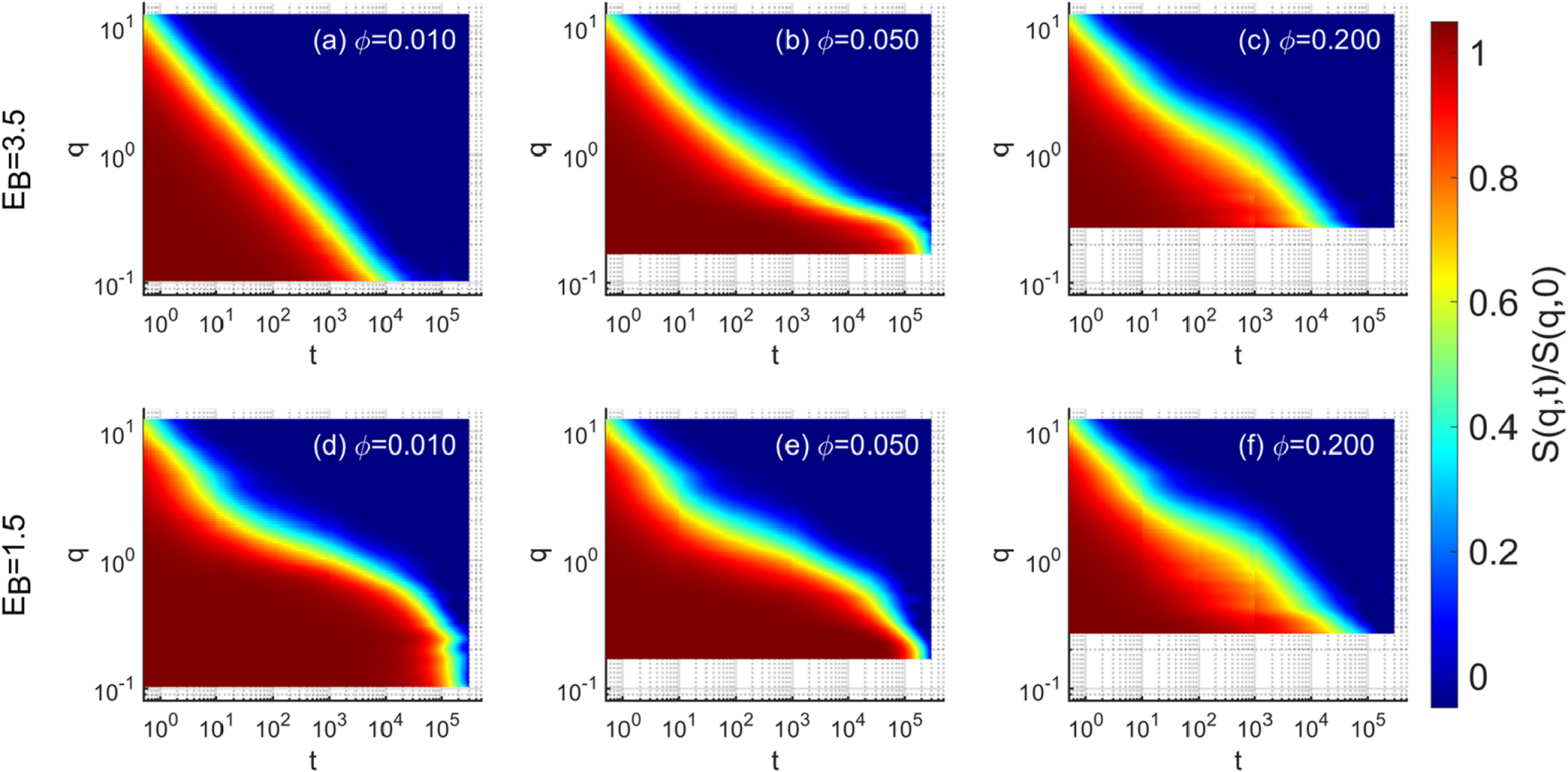
*q*-dependent dynamic structure factor *S*(*q*,*t*)/*S*(*q*,0) of the selective systems. (a, b) are for the systems with *E*_B_ = 3.5. (c, d) are for the systems with *E*_B_ = 1.5. When the bonding reaction is weak, such as in (a, b), the decaying pattern of the DSF is similar to that of a simple liquid (see Figure S15 in the Supporting Information). Otherwise, the decays are significantly extended except at the small length scale (large *q*), suggesting the deceleration in the relaxation because of the formation of clusters and networks.

The *q*-dependent characteristic times τ_*c*_ for the *E*_B_ = 3.5 and *E*_B_ = 1.5 systems are summarized in Figure 9a,b, respectively. The open symbols represent the τ_*c*_ = τ_c_^*^Γ­(1/β)/β results based on the DSF decay fitted against the stretch exponential *S*(*q*,*t*)/*S*(*q*,0) = exp (−(*t*/τ_c_^*^)^β^). When *E*_B_ = 3.5 and ϕ is low, the simple liquid-like behavior, e.g., τ_*c*_ ∼ *q* ^–2^ is observed. As ϕ approaches the threshold of percolation, the τ_*c*_ ∼ *q* ^–2^ scaling prediction fails as the τ_*c*_ results significantly deviate from the simple liquid limit (black dashed lines). Specifically, τ_*c*_(*q*) is about 1 order of magnitude greater than the simple liquid limit with the same *q* when *q* < 1.0. Such deviations of the *q*-dependent relaxation time indicate a strong nonlocal effect of the percolated particle networks. A higher ϕ results in a greater magnitude of deviation. Additionally, the transition zones, i.e., where τ_*c*_ starts to increase, shift rightward as ϕ increases. For the ϕ = 0.2 case, the transition starts at a small length scale as *q* ≈ 10.0, suggesting the percolation significantly decelerates the relaxations of the NPs at all length scales larger than the diameter of the NPs. When *E*_B_ = 1.5, the τ_*c*_ in the low-*q* regime is increased by almost 2 orders of magnitude for most cases, confirming that *E*_B_ = 1.5 indeed drives stronger bonding reactions. It is worth noting that the orders-of-magnitude increase of τ_*c*_ is also observed in some XPCS studies.^79,80^ More interestingly, a τ_*c*_ ∼ *q* ^–4^ scaling emerges in the transition zones for the systems with strong bonding reactions.^81^ Although the agreement is excellent, a deep understanding regarding the nonlocal effect is not established by these experimental studies. In Figure 9, there are local peaks at the length scale of a NP (*q* ≈ 2π), suggesting that the strong bonding reactions induce a collective effect on the relaxation of NPs. Such a collective effect is also observed in experiments by Yavitt et al.^57^ In their work, the mode-coupling theory (MCT) prediction^82^ was applied to quantify the collective effect. Therefore, the MCT prediction is also provided here, and the results are represented by the solid lines, as shown in Figure 9. See the Supporting Information for the details of MCT prediction. However, MCT only qualitatively predicts the *q*-dependent τ_*c*_ and positions of the local peaks, and is not able to directly quantify the nonlocal effects observed in Figure 9. For the systems with *E*_B_ = 1.5 and medium-ϕ, the MCT overestimates the τ_*c*_ in the small-*q* (*q* ≈ 0.3) and large-*q* (*q* ≈ 3.0) regimes, while underestimates at the medium-*q* (*q* ≈ 1.0) regime. On the other hand, for the low- and high-ϕ cases, the MCT prediction seems to underestimate the τ_*c*_ significantly. It is worth noting that the positions of the local peaks estimated here are close to *q* ≈ 4.0 instead of *q* ≈ 2π shown in the experimental measurement.^57^ For the systems with *E*_B_ = 3.5, the MCT prediction significantly underestimates the τ_*c*_ for all cases. Although the MCT predictions match the τ_*c*_ results at around *q* ≈ 10.0 for the high-ϕ cases, the predictions for the low-ϕ cases notably deviate by at least 1 order of magnitude, since the MCT predictions are similar to the simple liquid limit. As MCT was originally developed for understanding the simple molecular and atomic systems^83^ by a mean field approximation, the long-range interactions (such as the networks in this work) are lumped together as a collective effect on the local atoms or molecules. Therefore, the MCT only predicts the *q*-dependent τ_*c*_ qualitatively. All results are shown in Figures S21–S25, where the predictions by the *Skold* approximation^84^ are also provided. The *Skold* approximation quantitatively predicts the τ_*c*_-*q* relations only at the low-*q* regime for the systems with high-ϕ.

**Figure 9:**
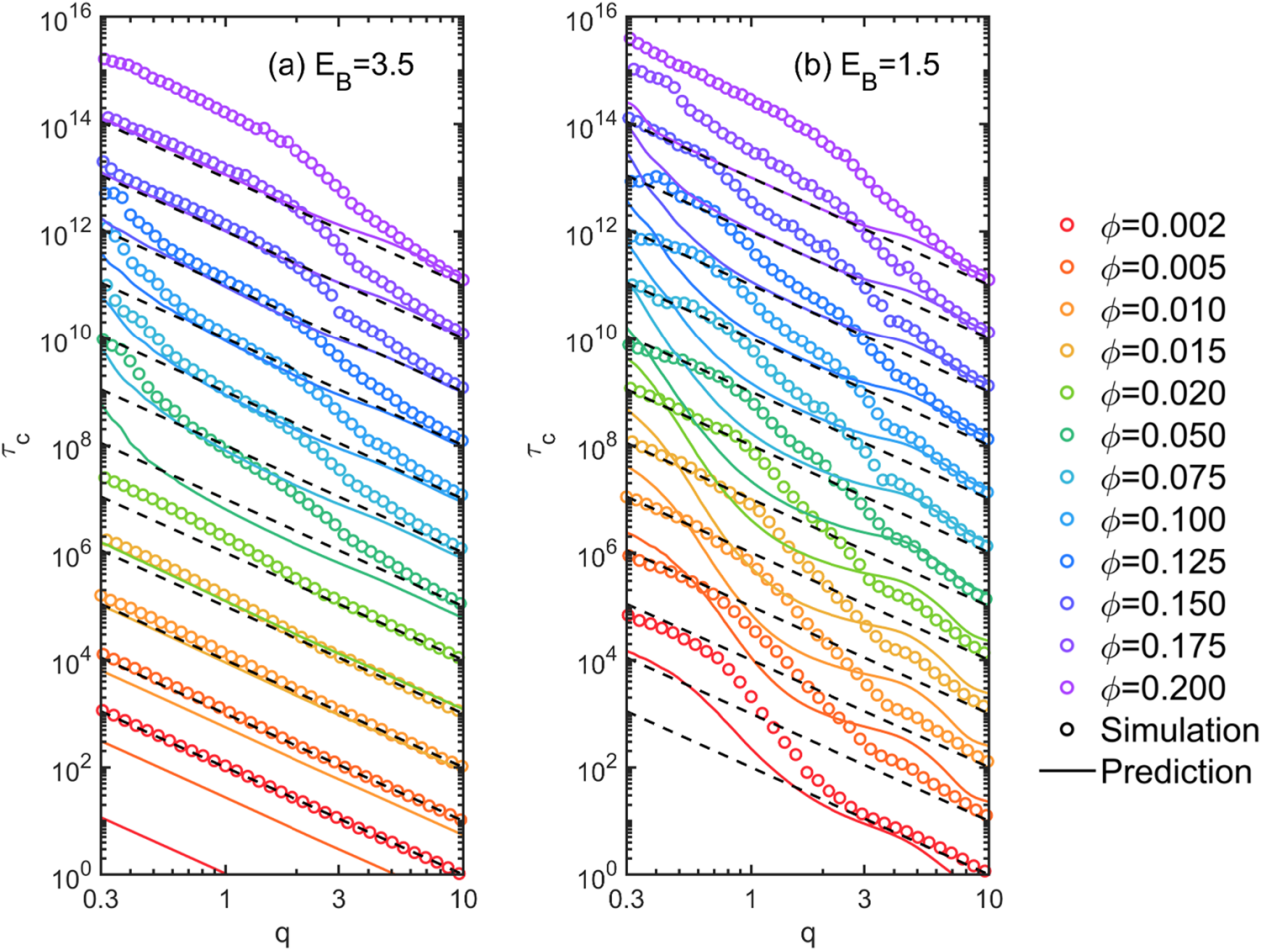
Characteristic time τ_*c*_ as functions of *E*_B_ and ϕ. (a) is for the systems with *E*_B_ = 3.5. (b) is for the systems with *E*_B_ = 1.5. The symbols represent simulation-based results. Solid curves are the prediction by the mode-coupling theory. Dashed lines represent the limit of a simple liquid. For visual clarity, all results are sequentially shifted as ϕ increases. The significant elevation of τ_*c*_ in the low-*q* regime suggests the deceleration in relaxation because of the clusters and networks. The local peaks at *q* ≈ 2π represent the collective effect.

### 3.5 Bulk Viscoelasticity

The bulk viscoelastic properties of the model PNCs are quantified by the relaxation modulus *G*(*t*) and nominal zero-rate viscosity η_0_. Specifically, *G*(*t*) is calculated according to eq 5. All *G*(*t*) results are shown in Figure S26. It is worth noting that the nonlinear reinforcement effect is clearly revealed by plotting *G*_0_ = *G*(*t*) as functions of ϕ and *E*_B_. Interestingly, the N.R. systems, i.e., without any bonding and debonding reactions, follows the Guth-Gold model that predicts *G*_0_ ∼ 2.5ϕ + 14.1ϕ^2^, as shown in Figure 10a.

**Figure 10:**
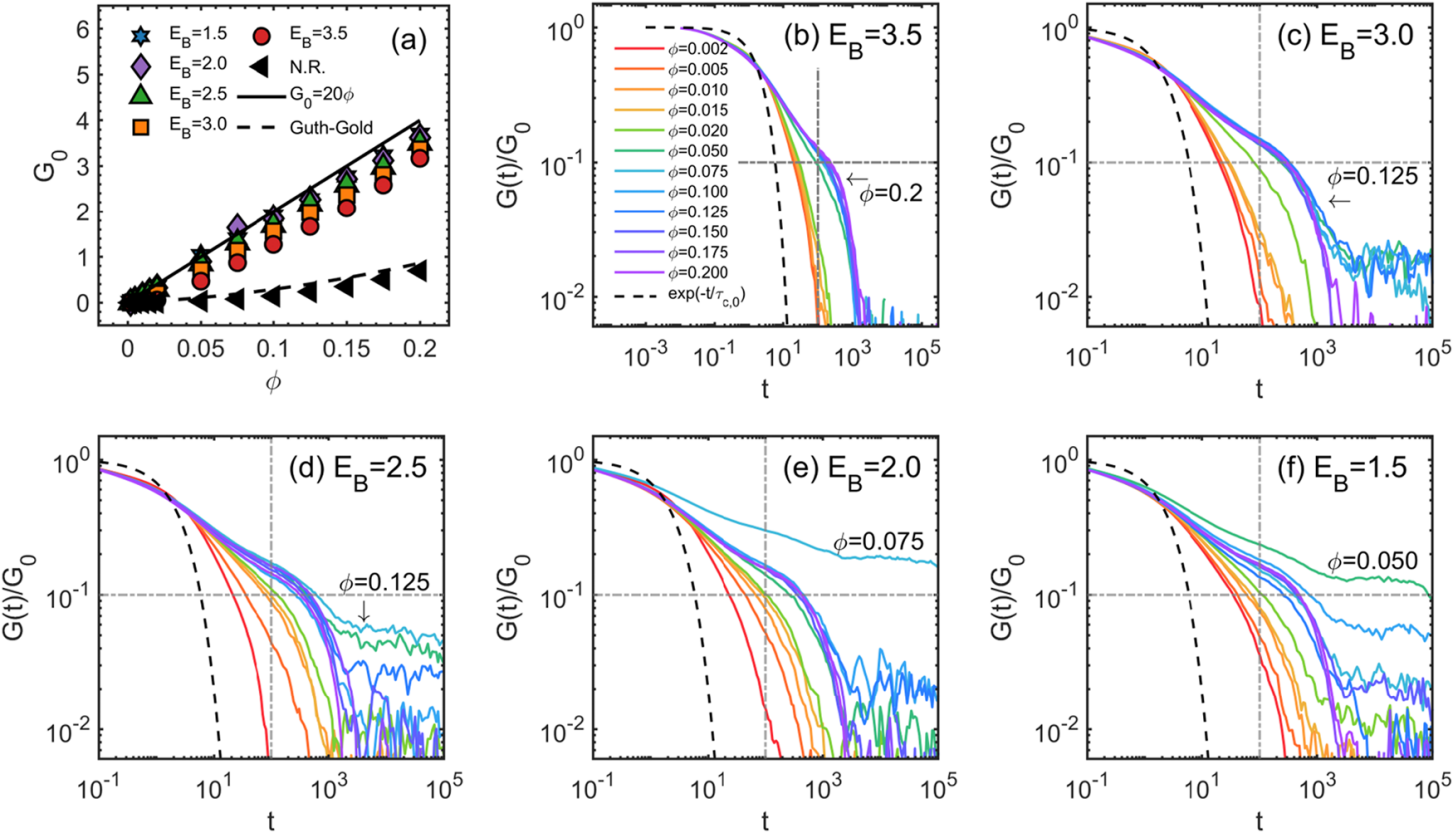
Relaxation modulus. (a) The magnitude of shear modulus *G*_0_ as functions of *E*_B_ and ϕ. While the results of the N.R. systems follow the Guth-Gold prediction, the model systems show a much stronger nonlinear reinforcement effect. (b–f) are for the systems with *E*_B_ = 3.5 to *E*_B_ = 1.5, respectively. The rescaled *G*(*t*) shows a strong dependency on *E*_B_ and ϕ. The magnitude of extension in the decay increases with ϕ but decreases with *E*_B_. For each *E*_B_, the most extended pattern is marked with the corresponding ϕ. The black dashed lines are for the limit of a simple liquid while the gray dot-dashed lines are for a guide to the eye.

The results of the N.R. systems underscore the hydrodynamic reinforcement in the absence of the bonding and debonding reactions. However, with active bonding and debonding reactions, the relaxation modulus magnitude *G*_0_ significantly increases with ϕ, as suggested by the simulation results that qualitatively follow the phenomenological *G*_0_ = 20ϕ trend. Additionally, a lower *E*_B_ results in a slightly higher *G*_0_, confirming that a lower *E*_B_ results in a stronger percolation effect. For a clear comparison of the decaying pattern of *G*(*t*), a renormalization by the corresponding *G*_0_ value is performed, and the results are shown in Figure 10b to f for the *E*_B_ = 3.5 to *E*_B_ = 1.5 systems, respectively. With the effective bonding reactions, the *G*(*t*) decays more slowly than the cases of a simple liquid that are shown in Figure S27, reflecting the decelerated microscopic relaxation in the former cases. When the bonding reaction is weak, e.g., *E*_B_ = 3.5 shown in in Figure 10b, the magnitude of deceleration is moderate. The low-ϕ systems share a similar decaying pattern. In comparison, the *G*(*t*) decays are even slower for the systems with ϕ ≥ 0.05, and a plateau emerges, suggesting the effect of the percolation. Such trends are shared by other percolated systems with different *E*_B_, and the level of deceleration increases with ϕ. For example, for the systems with *E*_B_ = 3.0 and *E*_B_ = 2.5, as shown in Figure 10c,d respectively, the plateaus of the high-ϕ systems cover a large range, and the decays are delayed. Additionally, the *G*(*t*) curves show a wider transition zone (near *t* = 10^2^) as ϕ increases. For the systems with *E*_B_ = 2.0 and *E*_B_ = 1.5, as shown in Figure 10e,f respectively, the plateau magnitudes are further increased and extended, confirming that stronger bulk viscoelasticity is correlated to lower *E*_B_. It is worth noting that *E*_*B*_ also affects the ϕ at which the most extended *G*(*t*) curves are identified, as explicitly indicated in the subpanels of Figure 10. A lower *E*_B_ results in a stronger bonding reaction. Therefore, a lower ϕ is correlated to a stronger percolation effect and thus to the most extended *G*(*t*) curve, as *E*_B_ decreases.

For a more direct comparison of the bulk viscoelasticity, the nominal zero-viscosity is calculated for all systems. The definition of zero-viscosity writes: η_0_ = ∫_0_^∞^ *G*(*t*)­d*t* for equilibrium simulation. However, the upper limit of the integral is nominally set to *t* = 100.0 here because the extended *G*(*t*) tails inevitably affect the numeric evaluation if *t* = ∞ is set for the upper limit. Another well-acknowledged treatment is to fit *G*(*t*) against phenomenological models such as the generalized Maxwell model and then to evaluate the integral analytically. However, it is not used here since the fitting may introduce extra uncertainty.

The estimated η_0_ results are shown in Figure 11. Like the *G*_0_ results, there is a strong dependency on ϕ and *E*_B_. In comparison, the N.R. systems follow the Guth-Gold model prediction. On the other hand, F.T. systems with permanent topology show the strongest nonlinear reinforcement. Please refer to Figure S28 for the results of low-ϕ systems. It is observed that a lower *E*_B_ results in increased η_0_, consistent with the discussion of other results that quantify the microscopic relaxation of the model polymer composites. Additionally, a phenomenological limit, i.e., η_0_(ϕ) = 300ϕ + 500ϕ^2^, is also shown for comparison. It is interesting that the viscosity of the system with ϕ = 0.05 and *E*_B_ = 1.5, and the system with ϕ = 0.75 and *E*_B_ = 2.0 are notably higher than the phenomenological limit because the most extended *G*(*t*) is not necessarily paired with the largest *G*_0_ magnitude as shown in shown in Figure 10. It is worth noting that the increase in η_0_ agrees well with experimental measurements of systems with different NPs and matrix polymers in terms of the nonlinear reinforcing effect.^85–88^ Establishing a more direct connection between the simulation results and experimental measurements will require further model extensions and parameter calibration, which are currently underway.

**Figure 11:**
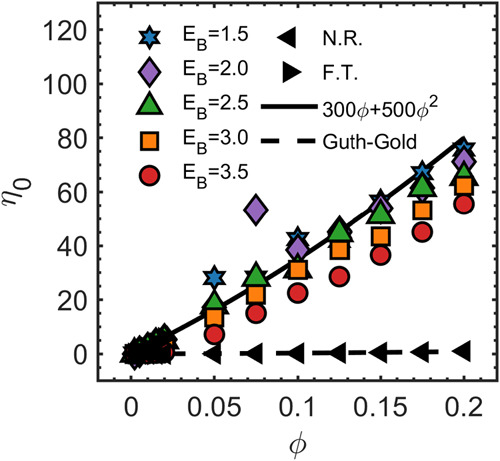
Zero-rate viscosity η_0_. The results of the N.R. and F.T. systems are included for a direct comparison. The N.R. systems follow the Guth-Gold prediction. All other systems follow the phenomenological relation marked by the solid line, suggesting significant nonlinear reinforcement. The zoom-in view of the low-ϕ regiem is shown in Figure S28 of the Suppporting Information.

### 3.6 Discussion

In the current work, we propose a mesoscale model that is used to investigate the viscoelasticity and nonlinear reinforcement of PNCs consisting of NPs embedded in a viscous liquid-like polymer-matrix. Since the basic length, mass, and energy scales *d*, *m*, and *k*_B_ *T* are set to unity in the model, for a reference system with spherical silica NP of 20.0 nm diameter at room temperature, for example, the unit mass and energy scale represent physical values of 5.54 × 10^3^ kg/mol and 2.47 × 10^3^ J/mol. Consequently, the characteristic time scale $∼\sqrt{md^{2}/k_{B}T}$ is about 29.8 ns, placing the proposed model in the mesoscale regime. Assume the matrix polymer is poly­(dimethylsiloxane) (PDMS) with *N*_*k*_ = 50 Kuhn segments whose molecular weight *M* is about 15.5 kg/mol, slightly above the entanglement threshold *M*_*e*_ = 12.3 kg/mol.^89^ Taking the Kuhn length *l*_*K*_ = 1.14 nm, the equilibrium end-to-end distance of the matrix PDMS is about *R*_ee_ = 8.1 nm, corresponding to the equilibrium distance *r*_2_ = 1.4 as *R*_ee_/*d* ≈ 0.4. Furthermore, the chain entropic stiffness is *K* = 3*k*_B_ *T*/*N*_*K*_ *l*_*K*_^2^ = 18.8 at *R*_ee_, agreeing with the results shown in Figure 1 semiquantitatively. This PDMS example illustrates the connection between our model parameters and the polymer physics. We believe that the proposed model is versatile and can be adapted for different types of PNCs representing diverse polymer and filler combinations of technological interest. The Yukawa potential is used to represent pairwise interaction between the NPs, resulting in a “soft” and repulsive behavior. The results of the rescaled radial distribution function (RDF) of systems with *E*_B_ = 1.5 are shown in Figure S29, where the minimum nonzero interparticle distance *r* ≈ 0.8 is smaller than unity, in contrast with what a hard-sphere/hard-wall potential would produce. It is suggested that there is no significant spatial overlapping between the NPs, which is consistent with the established physical understanding of PNCs. Alternatively, using the classical Weeks–Chandler–Andersen (WCA) potential would enforce a purely repulsive interaction between the particles. However, such a choice of the pairwise potential would induce numerical instability for simulations with our current choice of time step *t* = 0.001. Reducing the time step may avoid such instability, but would significantly increase the computational cost. Therefore, such a simulation setting is not adopted, considering our goal of investigating long-term dynamics of the model PNCs. Pairwise potentials with attractive tails and/or long-range weak forces can be used to model NPs with attractive interactions, such as charged particles.^90^ The proposed mesoscale model can be generalized and applied to model polymer-grafted NPs. If the chemical details of the grafting polymers are of interest, they should be explicitly incorporated; otherwise, implicit representations should be considered, such as tuning the friction coefficient through the thermostat or modifying the parameters of the bonding and debonding reactions.

Regarding the operational formulation of the bonded interaction, four equilibrium distances are used for the multi-Gaussian potential to balance the computational cost and the physical representation of the bridging chains. In fact, increasing the number of equilibrium distances, and thus the number of local stiffness and debonding energy barriers, may extend the capability of the mesoscale model. For example, in the continuous limit, the bonded interaction can represent the glass transition of the bound layer as demonstrated in the literature.^60,61^ However, the computational cost and the number of parameters would increase significantly. Since our goal is to probe network-like behavior of NPs rather than understand properties of the bound layer, we used only four equilibrium distance values. In terms of the maximum number of bonds that an NP can have, it is shown that bonding reactions of the studied systems remain below saturation levels. In fact, for systems with the highest degree of NP-NP bonding, i.e., for *E*_B_ = 1.5 and ϕ = 0.2 only 85% of possible bonds were generated at the end of the equilibration stage and remained statistically unchanged during the whole production run Systems with higher *E*_B_ have even lower degree of reaction. Therefore, it is believed that our choice of *n*_max,*b*_ = 5 is sufficient for capturing all network-like behavior and that increasing *n*_max,*b*_ will not affect the conclusions of this work. Additionally, the volume fraction ϕ effectively spans a wide range both below and above the percolation threshold, enabling a systematic investigation of the relaxation and viscoelasticity of model PNCs. The effect of the polymer matrix is implicitly modeled by overdamped Langevin dynamics with a given friction coefficient ξ. Such a choice assumes that the polymer matrix is like a simple viscous liquid and therefore the model does not account for complex effects of entanglement or cross-linking of the polymer matrix. Additional degrees of freedom can be integrated into the proposed model, if the entanglement and cross-linking effects are of interest. For example, adding explicit slip-links can represent the bridging chains between the NPs to enforce the physical uncrossability of entanglement.^91–93^ Similarly, if the heterogeneous interfacial segmental dynamics are of interest, it is valuable to incorporate an explicit Kuhn representation for the bridging chains. Additionally, the adapted overdamped Langevin dynamics enforces liquid-like behavior such that the background matrix is assumed at a temperature much higher than *T*_g_ (melt state). A more complex representation of the bridging chains is needed if the glassy relaxation is of interest.^61^ The inclusions of explicit representations may significantly affect the viscoelasticity of PNCs and thus are worth further investigation. However, the inclusions require additional computational cost, necessitating a careful balance between computational efficiency and simulation fidelity. It is also assumed that the bulk polymer matrix away from the NPs remains unperturbed, consistent with established physical understanding of PNCs.^7,94^ Therefore, only the contributions from the bonded and pairwise interactions between NPs are considered in our model, with effects from the polymer matrix considered at an implicit level.

It is also worth noting that the bonding and debonding reactions are collectively controlled by the parameters *E*_B_, *E*_DB_, τ_MC_, *p*_MC_, and *l*_0_. Except for *E*_B_, the other parameters are fixed for all simulations. The parameters τ_MC_ and *p*_MC_ control the bonding and debonding reactions linearly as they are only prefactors when determining if the reactions are active or not. However, *E*_B_, *E*_DB_, and *l*_0_ have a nonlinear effect on the reactions since they are in the exponential expressions of the MC steps. For example, a lower probability of debonding reaction is controlled by a higher *E*_DB_, and therefore glassy bridging chains can be represented by bonds with *E*_DB_ values significantly higher than the thermal energy *k*_B_ *T*. A low *E*_B_ value implies a more probable bonding reaction, which corresponds to an attractive polymer-NP interaction. On the other hand, a higher *E*_B_ represents a repulsive polymer–particle interaction as the bonding reaction is not favored. Indeed, the relaxation dynamics and viscoelasticity of the PNCs system significantly change as *E*_B_ is decreased from 3.5*k*_B_ *T* to 1.5*k*_B_ *T*, with an increasing percolation that is driven by stronger polymer–particle interaction. Overall, relatively low *E*_B_ values are adapted to avoid complex behavior such as glass transition and chemical cross-linking, for the purpose of model development and demonstration. The dynamic bonds represent possible relaxation of the bridging chains. Therefore, the proposed model and presented results are intended to be correlated with experimental studies in which the polymer matrices have low molecular weight and are in the rubbery state. Additionally, the equilibrium distances *r*_*i*_ and local stiffness *K* of the bonded interaction collectively represent the structural properties of the bridging chains, such as chain length and stiffness. By fine-tuning these parameters, the proposed model can be calibrated against chemistry-specific systems of interest. It is also worth noting that the application of the proposed model in nonequilibrium simulation is straightforward, and can be valuable in systematically investigating nonreversible behavior of PNCs, such as the Mullins effects.^60^ Such application will enable direct quantification of the network structure that is subject to the loading history without the need to introduce phenomenological variables such as the degree of internal damage that is widely adopted by continuum modeling.^95,96^

Most importantly, the nonlocal effect on the viscoelasticity of our model PNC is evidently supported by the analyses of dynamic structure factor and the characteristic time τ_*c*_. First, the comparison between Figures 8 and S14, and between Figures 9 and S15, clearly shows the network-induced effect due to the bonding and debonding reactions, in contrast to simple liquid-like behavior. Simple liquid-like relaxation dictates the local effect on viscoelasticity, as suggested by comparing simulation results (symbols) and the simple liquid limit (dashed lines) in Figure 9. Second, we performed the analyses based on the mode-coupling theory (MCT). While the MCT prediction captures the local peak in the large-*q* regime relatively well, it fails to quantify the mid- and small-*q* behavior. This nonlocality governs the nonlinear reinforcement, as suggested by the results of relaxation modulus and zero-rate viscosity.

In terms of the modified Rouse model, there is no rebonding process as present in the mesoscale simulations. Therefore, the MSD curves eventually converge to the diffusive limit of the simple liquid as the effect of the spring constant *k*(*t*) vanishes. Linking the modified Rouse model to the prediction of the bulk viscoelasticity is out of the scope of the current work, but it will be a goal of our future research. In terms of the characteristic relaxation time τ_*c*_, it should be noted that no normalization was applied to the MCT predictions of Figure 9. In fact, when we performed normalization following a procedure presented in the literature,^57^ the MCT predictions matched the simulation results with higher quantitative accuracy. However, asserting such apparent consistency may lead to an inaccurate presentation of the results and implications. Last but not least, the ∼2.5ϕ + 14.1ϕ^2^ expression is used for the Guth-Gold prediction in this work instead of the original expression^5^ for the *G*_0_ and η_0_ results.

It is worth noting that the volume fraction ϕ = 0.05 is identified as the percolation threshold under the consideration of the effect of particle clustering and networking on the viscoelasticity. The significant change in dynamic properties, e.g., the MSD and decay of relaxation modulus, is closely related to the apparent formation of the infinitely large network across the periodic boundaries. At the same time, while ϕ ∼ 0.05 appears to be a good semiquantitative estimate of percolation threshold, we recognize that an exact threshold limit may be affected by simulation details, such as the size of periodic box size, and other model parameters, such as the strength of correlation.^97^ As an attempt to overcome the finite-cell-size effects we have pushed simulations with the number of NPs (*N*_total_) as large as 4 × 10^4^ (corresponding to physical cell size in the range of 950 to 4400 nm). However, a thorough investigation of the dependence of percolation threshold on all factors warrants a dedicated study on its own and is beyond the scope of the present work.

## 4 Conclusion

In this study, a mesoscale model is developed to investigate the viscoelasticity and nonlinear reinforcement of model PNCs. Specifically, each individual NP is modeled as an isotropic bead, and the effect of bridging polymer chains between different NPs is represented by a bonded interaction with a multi-Gaussian potential. Equilibrium dynamics simulations are performed based on the overdamped Langevin dynamics with a given friction coefficient ξ. The particle volume fraction ϕ is systematically varied. Additionally, the bonding and debonding reactions are modeled by the Bell model and implemented in a Metropolis Monte Carlo algorithm, which generates and annihilates NP-NP bonds according to given energy barriers *E*_B_ and *E*_DB_. Extensive simulations are performed to generate the equilibrium configurations of clusters and percolated networks. Analyses of structural and dynamic properties reveal that both ϕ and *E*_B_ strongly affect the viscoelastic properties of the model PNCs. Particularly, ϕ = 0.05 is identified as the threshold of percolation. The radius-of-gyration and static structure factor confirm that percolated networks are formed when ϕ ≥ 0.05. Otherwise, most of the particles remain free-standing or in clusters of limited size. The microscopic characteristics of the bonding and debonding reactions are quantified by the bond lifetime τ_*b*_ that is dependent on ϕ and *E*_B_. In the bulk, the mean-squared displacement analysis suggests that ϕ and *E*_B_ essentially control the diffusive behaviors. A modified Rouse model is proposed to predict the diffusion coefficient *D*. More importantly, the *q*-dependent characteristic relaxation time τ_*c*_ reveals that the percolated networks of particles dictate the viscoelasticity and nonlinear reinforcement of the model PNCs, which is confirmed by the investigation of the relaxation modulus and zero-rate viscosity. Most significantly, network-like structures emerge at length scales much larger than individual NPs that significantly decelerate the relaxation of the NPs, a phenomenon not been highlighted enough in previous simulation studies. Such deceleration is much stronger than the mean field MCT prediction, which only quantifies a collective behavior at the length scale similar to that of single NPs. The observed nonlinear reinforcement of the model PNCs originates from this network-induced deceleration in particle relaxation. The general applicability of the proposed mesoscale model provides a powerful framework for studying the viscoelasticity and nonlinear filler reinforcement of a wide range of PNCs.

## Supplementary Material



## Notes

Details of the modified Rouse model and MCT prediction, additional results: simulation snapshot; relation between radius-of-gyration and clusters size; static structure factor; characteristics of the bond reaction; mean-squared displacement; diffusion coefficient; relation between the mean-squared displacement and cutoff; modal mean-squared displacement of the classical Rouse model; relation between diffusion coefficient and characteristic time of the spring in the modified Rouse model; dynamic structure factor and its characteristic time as functions of the length scale; relaxation modulus; viscosity; radial distribution function (PDF)
