# Supplementary material for: Nonlocal Effect of Percolated Particle Networks on Viscoelasticity of Polymer–Filler Nanocomposites: A Mesoscale Simulation Study
Source: Macromolecules. 2026 Jun 3;59(12):6676–91. doi: 10.1021/acs.macromol.6c00792 (PMC13296481; doi:10.1021/acs.macromol.6c00792)
Supplement: Supplementary file 1 [file ma6c00792_si_001.pdf]

Supporting Information to

Nonlocal Effect of Percolated Particle Networks on Viscoelasticity of Polymer—filler Nanocomposites: A Mesoscale Simulation Study

Weikang Xian,[1] Zihan Tang,[1] Amitesh Maiti,[2] Andrew P. Saab,[2] and Ying Li[1,]*

[1] Department of Mechanical Engineering, University of Wisconsin-Madison, Madison, WI 53706-1572, USA

[2] Lawrence Livermore National Laboratory, Livermore, California 94550, USA

E-mail: yli2562@wisc.edu

S1. Methods

S1.1. Modified Rouse Model. The detailed derivation of the modified Rouse model and its link to the mean-squared displacement (MSD) are provided here. For a cluster of nanoparticles (NPs), the topology is fully described by its adjacency matrix $\boldsymbol{A}$ or Rouse-Zimm matrix $\boldsymbol{Z}$, which is a function of $\boldsymbol{A}$. With the assumption that the cluster is immersed in a viscous environment and subject to random dissipation, its relaxation follows the overdamped Langevin dynamics, as in **eq S1,** where $\boldsymbol{R}$ is the coordinates of the NPs, i.e., R_i with $i = 1 \cdots N$. ξ is the friction coefficient specific to an individual degree-of-freedom, $k(t)$ is the elastic constant of the bond between two NPs. D is the diffusion coefficient of a free NP as given by $D_0 = k_B T/\xi$. $\boldsymbol{W}(t)$ are the vectorized Wiener processes. Let's define $\eta(t) = \sqrt{2k_B T\xi}\, dW/dt$. According to the fluctuation-dissipation theorem, one gets $\langle \eta_i(t)\eta_i(t')\rangle = 2k_B T\xi\delta_{ij}\delta(t-t')$, which is also often used to derive **eq S1**.

$$dR = -\frac{k(t)}{\xi} ZRdt + \sqrt{2D_0}dW(t) \quad \text{eq. S1}$$

Since Z is symmetric, it can be diagonalized by its eigenvalues Λ and normalized eigenvectors V, and writes $Z = V\Lambda V^{\mathrm{T}}$. Note that $I = VV^{\mathrm{T}} = VV^{-1}$. The operation $X = V^{\mathrm{T}}R$ linearly maps the coordinates in the Cartesian space to the normal-mode space. The components of X, X_p with $p = 0 \cdots N-1$, are orthogonal to each other. Substitute $R = VX$ into **eq S1**, one gets **eq S2**

$$dX = -\frac{k(t)}{\xi} \Lambda Xdt + \sqrt{2D_0}dW_p(t) \quad \text{eq. S2}$$

of which individual components are decoupled from each other due to the diagonalization of Λ. Note that $W_p(t) = V^{\mathrm{T}}W(t)$ are also vectorized Wiener processes with the independent components. Please refer to ***Note 1*** for more details. For each mode, as in **eq S3**

$$dX_p = -a(t)X_p dt + \sqrt{2D}dW_p(t) \quad \text{eq. S3}$$

where $a(t) = \lambda_p k(t)/\xi$. Let's define an integration factor $A(t)$ and an auxiliary function $B(t)$, as respectively in **eq S4** and **eq S5**,

$$A(t) = \int_0^t a(s)ds \quad \text{eq. S4}$$

$$B(t) = e^{-A(t)} \quad \text{eq. S5}$$

Multiply both sides of **eq S3** by $A(t)$ yields **eq S6**

$$d\left(e^{A(t)}X_p\right) = e^{A(t)}\sqrt{2D_0}dW_p(t) \quad \text{eq. S6}$$

Integrating **eq S6**, from 0 to t, and rearranging yields the evolution of the individual modes, as in **eq S7**

$$X_p(t) = B(t)X_p(0) + \sqrt{2D_0}B(t)\int_0^t \frac{1}{B(s)}dW_p(s) \qquad \text{eq. S7}$$

Moreover, it is convenient to define the second term on the right-hand side of **eq S7** as $Y(t)$. Note that its expectation is $E[Y(t)] = 0$, and variance is $Var[Y(t)] = E[Y^2(t)]$. Please refer to ***Note 2*** for more details. Importantly, **eq S7** is connected to the mean-squared displacement function $g(t)$, as in **eq S8**

$$g(t) \equiv \frac{1}{N}E[\|\boldsymbol{R}(t) - \boldsymbol{R}(0)\|] = \frac{1}{N}E[\|\boldsymbol{X}(t) - \boldsymbol{X}(0)\|] \qquad \text{eq. S8}$$

in which $\|\boldsymbol{R}\| = \boldsymbol{R}^T\boldsymbol{R}$ is the vector-norm operation. Specifically, the expectation of an individual mode is given in **eq S9**

$$E\left[\left(X_p(t) - X_p(0)\right)^2\right] = (B(t) - 1)^2X_p^2(0) + 2D_0B^2(t)\int_0^t \frac{1}{B^2(s)}ds \qquad \text{eq. S9}$$

Therefore, for a cluster of NPs, its MSD can be analytically derived according to its $\boldsymbol{Z}$ and initial coordinates $\boldsymbol{R}(0)$, assuming zero-velocity conditions. In the case of permanently bonded spring[1], i.e., $k(t) = k_0$ and $a_0 = \lambda k_0/\xi$. Additionally, $A(t) = a_0t$ and $B(t) = e^{-a_0t}$. Therefore, the expectation of each mode writes specifically, as in **eq S10**

$$E\left[\left(X_p(t) - X_p(0)\right)^2\right] = (e^{-a_0t} - 1)^2X_p^2(0) + \frac{D_0}{a_0}(1 - e^{-2a_0t}) \qquad \text{eq. S10}$$

It is worth noting that a free-standing cluster, there is always a zero-eigen value that corresponds to the diffusive MSD of the center-of-mass of the cluster. In such a special case, the first term of

the right-hand side in **eq S10** vanishes, and the second term degenerate to $2Dt$, considering the Taylor expansion of $e^{-2a_0 t} = 1 - 2a_0 t + O(a_0^2)$. A summation of X_p in all three directions yields the relation of $g(t) = 6D_0 t/N$, as expected for the free-diffusion limit.

In the case of time-dependent spring whose lifetime exponentially decays, i.e., $k(t) = k_0 e^{-t/\tau_k}$. As $a(t) = a_0 e^{-t/\tau_k}$, the integration factor writes $A(t) = a_0 \tau_k \left(1 - e^{-t/\tau_k}\right)$, and $B(t) = \exp\left[-a_0 \tau_k \left(1 - e^{-t/\tau_k}\right)\right]$. Therefore, the expectation of each mode writes specifically, as in **eq S11**

$$E\left[\left(X_p(t) - X_p(0)\right)^2\right] = (e^{-a_0 t} - 1)^2 X_p^2(0) + 2D_0 B^2(t) I(t) \qquad \text{eq. S11}$$

where $I(t) = \tau_k e^{2a_0 \tau_k}\left[E_1\left(2a_0 \tau_k e^{-t/\tau_k}\right) - E_1(2a_0 \tau_k)\right]$, and $E_1(x) = \int_x^\infty \frac{e^{-t}}{t} dt$ is the exponential integral. Please refer to ***Note 3*** for more details of evaluating $I(t)$. It should be noted that in the limit of $\tau_k = \infty$, **eq S11** degenerates to **eq S10**.

***Note 1*. Wiener Processes in the Cartesian and the Modal Spaces**

For the vectorized Wiener processes, $\boldsymbol{W_p}(t)$, in the modal spaces, it can be written as

$$dW_p(t) = \sum_{i=1}^{N} V_{ip}\, dW_i(t)$$

where V_{ip} is the components of the eigenvectors. Its expectation is given by

$$E\left[dW_p(t)\right] = E\left[\sum_{i=1}^{N} V_{ip}\, dW_i(t)\right] = \sum_{i=1}^{N} V_{ip}\, E[dW_i(t)] = 0$$

as $E[dW_i(t)] = 0$ is an intrinsic property of the random process. Additionally, the variance by definition is $Var[dW_p(t)] = E[d^2W_p(t)] - (E[dW_p(t)])^2$. As the second term on the right-hand side is zero, the variance equals to $E[\delta_{pq}dW_p(t)dW_q(t)]$, which writes

$$E[\delta_{pq}dW_p(t)dW_q(t)] = E\left[\delta_{pq}\sum_{i=1}^{N} V_{ip}\, dW_i(t)\sum_{j=1}^{N} V_{jq}\, dW_j(t)\right]$$

Rearranging the right-hand side yields

$$\delta_{pq}E\left[\sum_{i=1}^{N} V_{ip}\sum_{j=1}^{N} V_{jq}\, dW_i(t)dW_j(t)\right] = \delta_{pq}\sum_{i=1}^{N}\sum_{j=1}^{N} V_{ip}V_{jq}\, E[dW_i(t)dW_i(t)]$$

Since $E[dW_i(t)dW_j(t)] = \delta_{ij}dt$

$$E[\delta_{pq}dW_p(t)dW_q(t)] = \delta_{pq}\delta_{ij}dt\sum_{i=1}^{N}\sum_{j=1}^{N} V_{ip}V_{jq} = dt\sum_{i=1}^{N} V_{ip}^2$$

Considering $\sum_{i=1}^{N} V_{ip}^2 = 1$ is an intrinsic property of the eigenvector $\boldsymbol{V}$, one gets $E[d^2W_p(t)] = dt$. In conclusion, the Wiener process in the modal space $\boldsymbol{W_p}(t)$ has the same properties as the Cartesian counterpart $\boldsymbol{W}(t)$.

***Note 2*. The Expectation and Variance of $Y(t)$**

For $Y(t) = \sqrt{2D}B(t)\int_0^t \frac{1}{B(s)}dW_p(s)$, it is straightforward that $E[Y(t)] = 0$ as the integration is regarding the $dW_p(s)$ of which the expectation is zero. Considering its variance, one writes the definition

$$Var[Y(t)] = E[Y^2(t)] - (E[Y(t)])^2$$

Since $E[Y(t)] = 0$, only the first term of the right-hand side is non-zero, therefore $Var[Y(t)] = E[Y^2(t)]$. Consider the following,

$$\frac{E\left[Y_p(t)Y_q(t)\right]}{2DB^2(t)} = \int_0^t \frac{1}{B_p(s)} \int_0^t \frac{1}{B_q(s)} E\left[dW_p(s)dW_q(s)\right] = \int_0^t \frac{1}{B_p(s)} \int_0^t \frac{1}{B_q(s)} \delta_{pq} ds$$

Therefore, one gets $Var[Y(t)] = E[Y^2(t)] = 2DB^2(t) \int_0^t \frac{1}{B^2(s)} ds$ and the orthogonality of $Y(t)$.

***Note 3*. The Evaluation of $I(t)$**

In the case of decaying bond with $k(t) = k_0 e^{-t/\tau_k}$. As $a(t) = a_0 e^{-t/\tau_k}$, one has $A(t) = a_0 \tau_k \left(1 - e^{-t/\tau_k}\right)$, and $B(t) = \exp\left[-a_0 \tau_k \left(1 - e^{-t/\tau_k}\right)\right]$. Therefore, the integral $\int_0^t \frac{1}{B^2(s)} ds$ in eq S9 is defined as $I(t)$ and specifically writes

$$I(t) = e^{2a_0\tau_k} \int_0^t \exp\left[-2a_0 \tau_k e^{-s/\tau_k}\right] ds$$

Define $u = e^{-s/\tau_k}$ such that $du = -\frac{1}{\tau_k} e^{-s/\tau_k} ds$ and $ds = -\frac{\tau_k}{u} du$. Substituting back and changing the integration limits, it yields

$$I(t) = \tau_k e^{2a_0\tau_k} \int_{e^{-t/\tau_k}}^{1} \frac{e^{-2a_0\tau_k u}}{u} du$$

Define $v = 2a_0 \tau_k u$ such that $du = \frac{dv}{2a_0\tau_k}$. Substituting back and changing the integration limits, it yields

$$\frac{I(t)}{\tau_k e^{2a_0\tau_k}} = \int_{2a_0\tau_k e^{-t/\tau_k}}^{2a_0\tau_k} \frac{e^{-v}}{v} dv = E_1\left(2a_0 \tau_k e^{-t/\tau_k}\right) - E_1(2a_0 \tau_k)$$

The "*expint*" function in MATLAB is used to evaluate the exponential integral $E_1(x)$.

S1.2. Mode-Coupling Theory. The mode-coupling theory (MCT) approximate the relaxation dynamics of the NPs by linking the diffusion to $S(q)$ that quantifies the microstructure of the collection of NPs.[2] Essentially, the q-dependent characteristic time is as in **eq S12**

$$\tau(q) = \frac{S(q)}{q^2 D_s}\left(1 + \frac{\rho}{16\pi^2}\int_0^{\infty} d\boldsymbol{k}\frac{|V_{\boldsymbol{k},\boldsymbol{q}-\boldsymbol{k}}|}{k^2/S(k) + (\boldsymbol{q}-\boldsymbol{k})^2/S(|\boldsymbol{q}-\boldsymbol{k}|)}\right) \qquad \text{eq. S12}$$

where $V_{\boldsymbol{k},\boldsymbol{q}-\boldsymbol{k}}$ is as in **eq S13** with $C_{\mathrm{eff}}(k) = 1/\rho - 1/\rho S(k)$, $q = |\boldsymbol{q}|$ and $\hat{\boldsymbol{q}} = \boldsymbol{q}/|\boldsymbol{q}|$.

$$V_{\boldsymbol{k},\boldsymbol{q}-\boldsymbol{k}} = (\hat{\boldsymbol{q}} \cdot \boldsymbol{k})C_{eff}(k) + \hat{\boldsymbol{q}} \cdot (\boldsymbol{q}-\boldsymbol{k})C_{eff}(|\boldsymbol{q}-\boldsymbol{k}|) \qquad \text{eq. S13}$$

Note that $S(\mathbf{k})$ is the static structure factor as described in the main text. ρ is the particle number density. The integral in **eq S12** is estimated by sampling by the $\boldsymbol{q}$ and $\boldsymbol{k}$ vectors uniformly in the reciprocal space. The upper limitation is effectively truncated at $q = 4\pi/L$ where L is the length of the simulation box. $D_s = D_0/C_n$ is the diffusion coefficient rescaled by the ϕ-dependent contact number C_n that is evaluated by integrating the radial distribution function up to $r = 1.1$. C_n effective accounts the number neighboring particles interacting with the center-probed particle, and D_s quantifies the effective diffusion subject to the interparticle interaction. $D_0 = 0.01$ is the diffusion coefficient of the NP-based simple liquid with no bonding and debonding reactions, as stated in the main text.

Additionally, the *Skold* approximation[3] to **eq S12** is also presented here for a comparison. The approximation is given in **eq S14**

$$\tau(q) = \frac{S(q)}{q^2 D_s}\left(1 + \frac{\rho}{16\pi^2}\int_0^{\infty} dq\, q^2 \frac{C_{eff}^2(q)S(k)}{1 + 1/S(q)}\right) \qquad \text{eq. S14}$$

The *Skold* approximation effectively simplifies the effect of multi-body interparticle interaction on the long-term relaxation.

S2. Additional Results

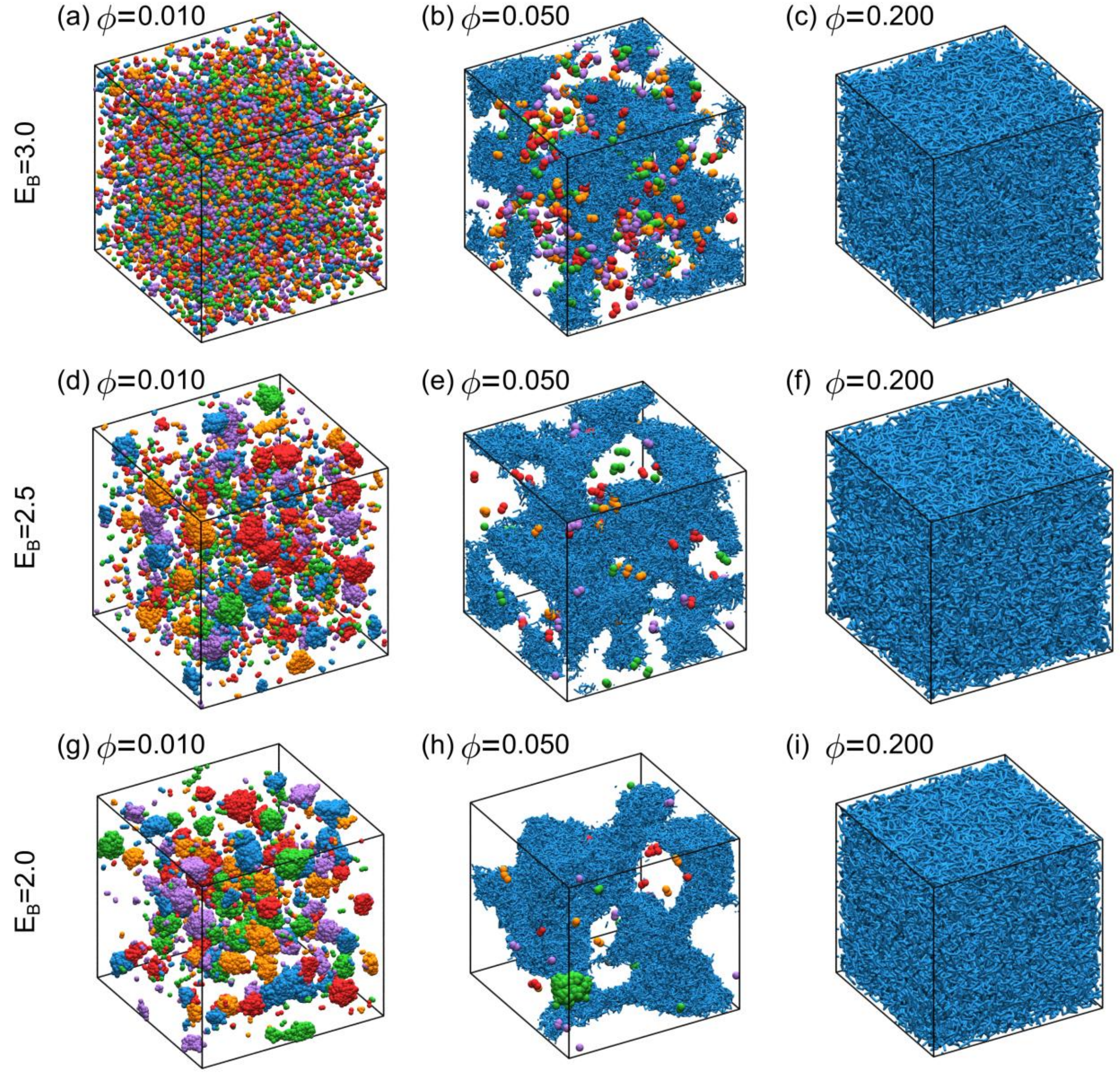

Figure S1. Selective snapshots of the simulation systems with $E_B = 3.0$ to $E_B = 2.0$.

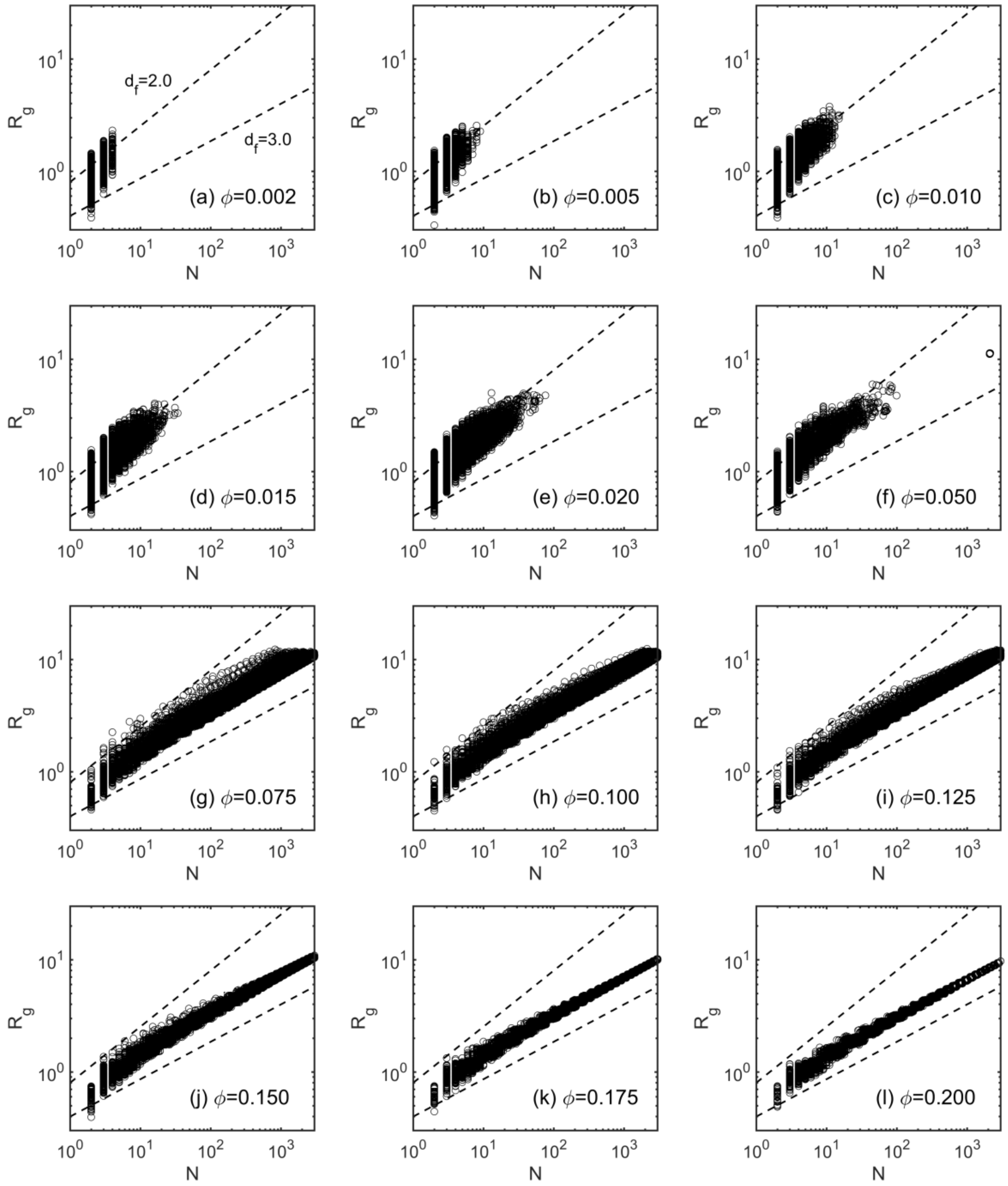

Figure S2. The R_g-N relations of system with $E_B = 3.5$.

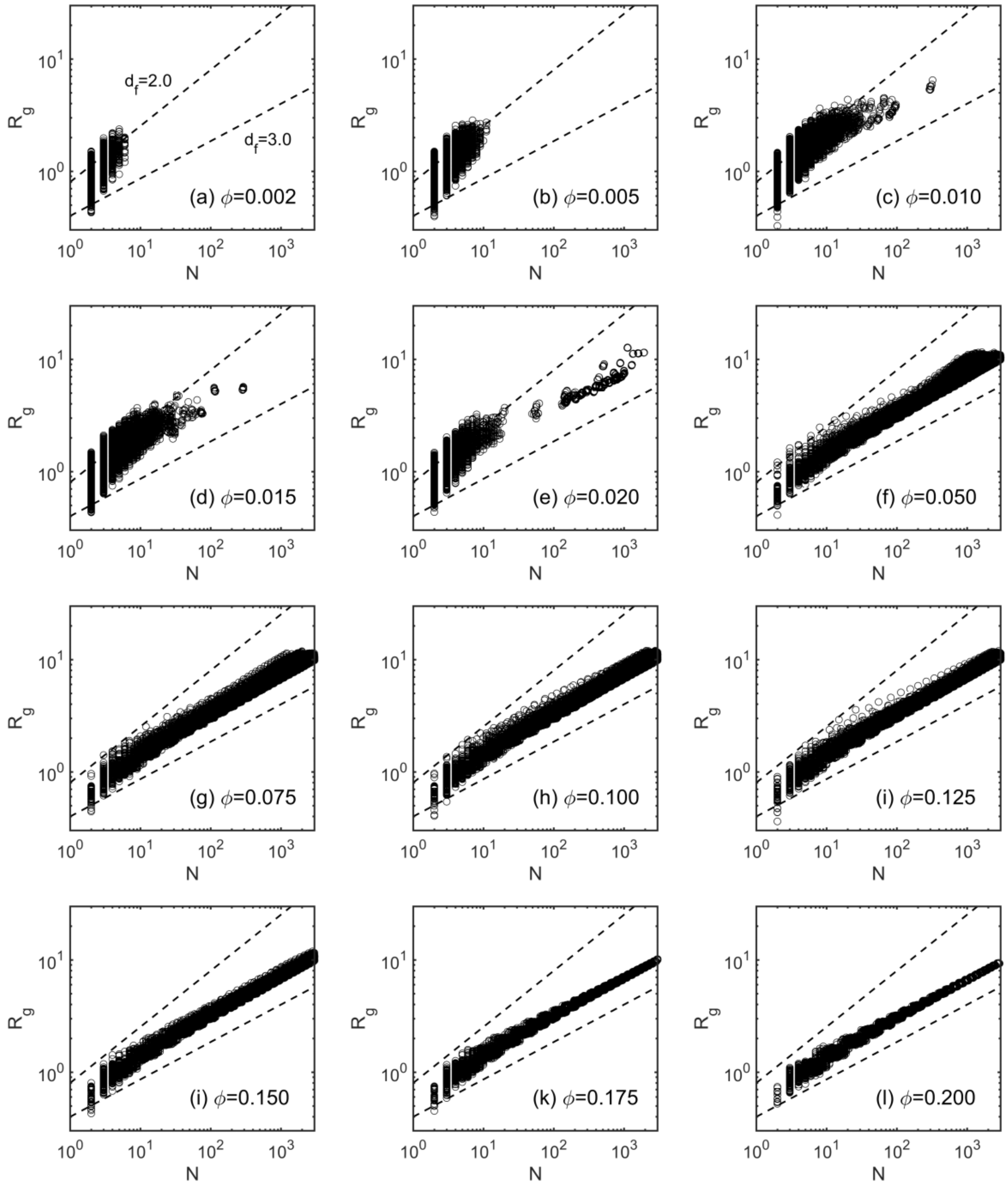

Figure S3. The R_g-N relations of system with $E_B = 3.0$.

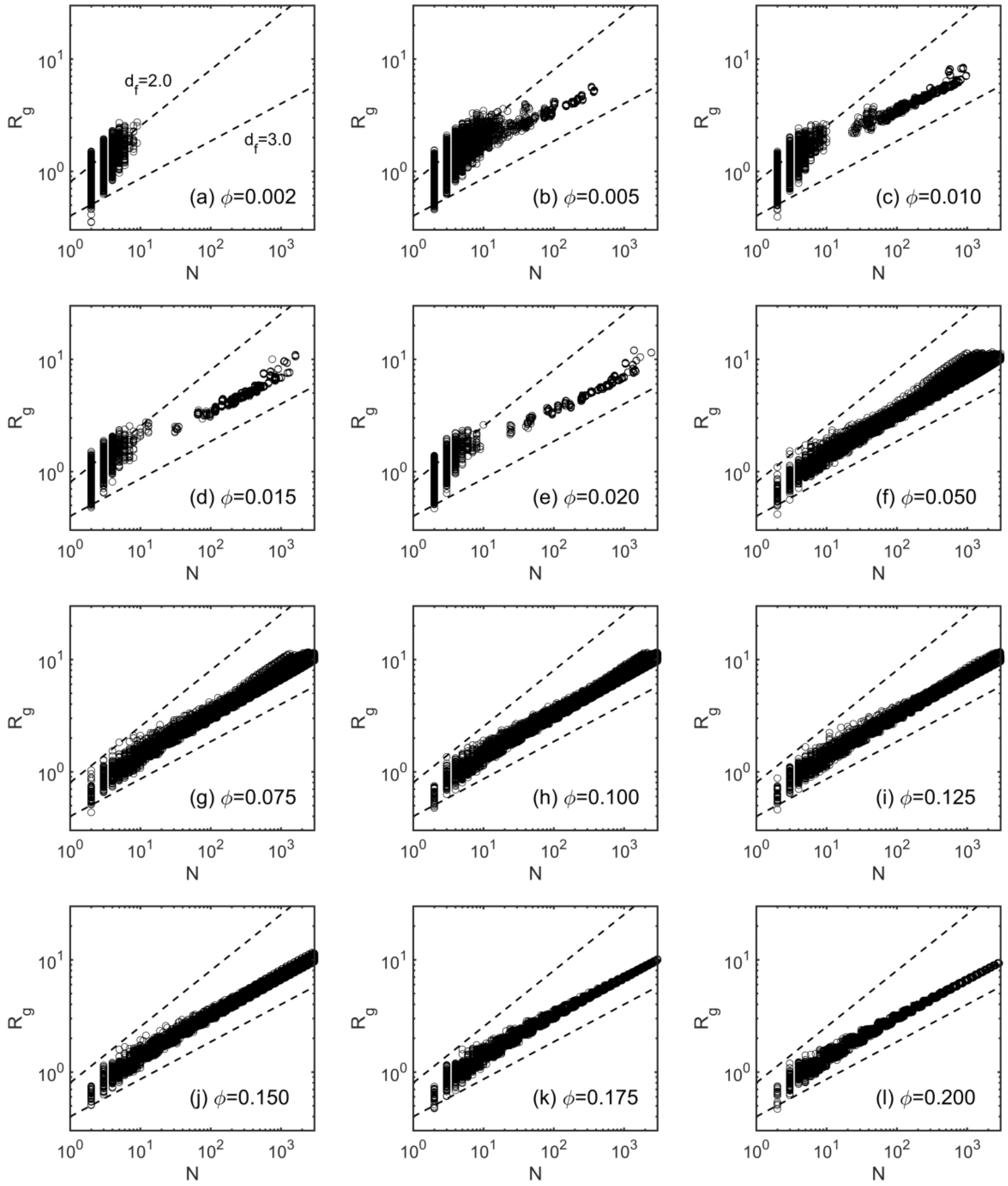

Figure S4. The R_g-N relations of system with $E_B = 2.5$.

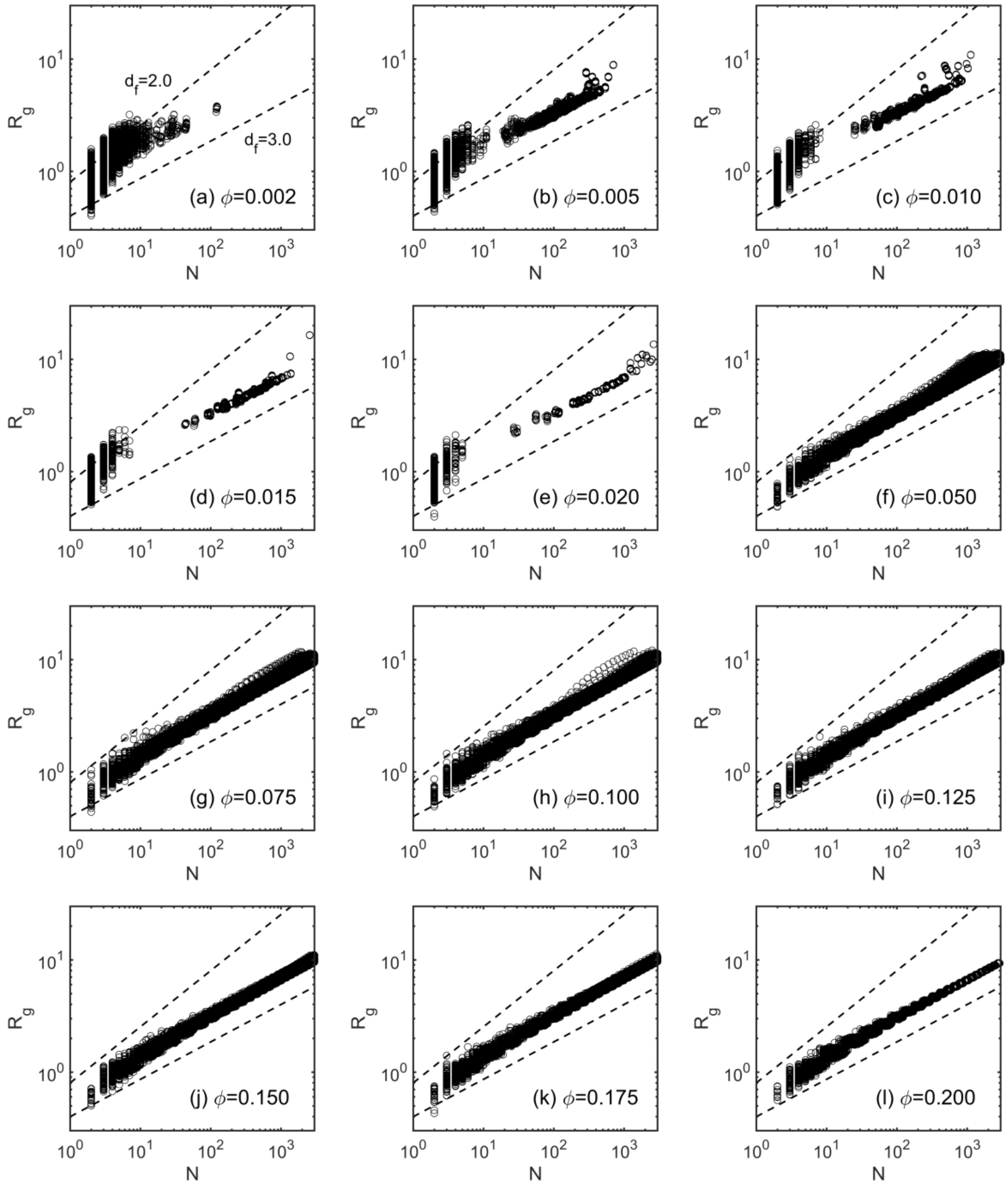

Figure S5. The R_g-N relations of system with $E_B = 2.0$.

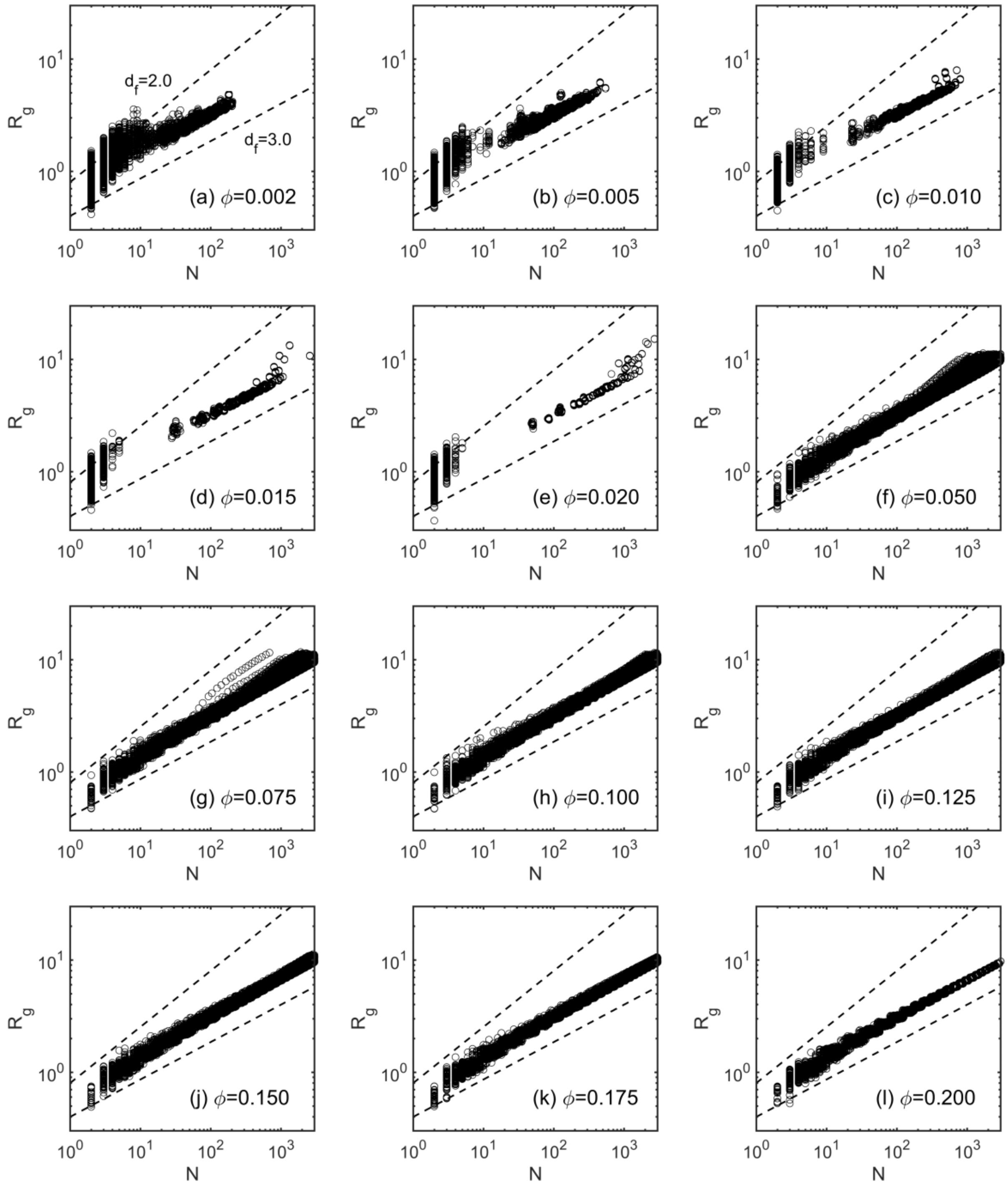

Figure S6. The R_g-N relations of system with $E_B = 1.5$.

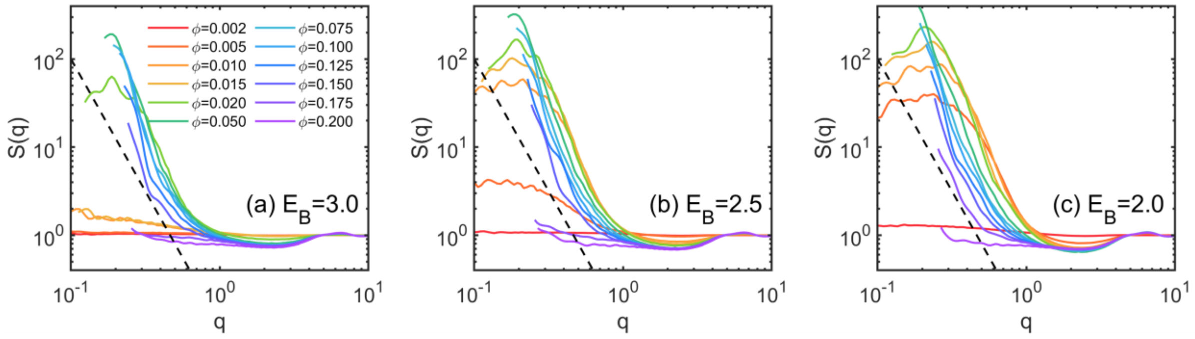

Figure S7. Static structure factors of the simulation systems with $E_B = 3.0$ to $E_B = 2.0$.

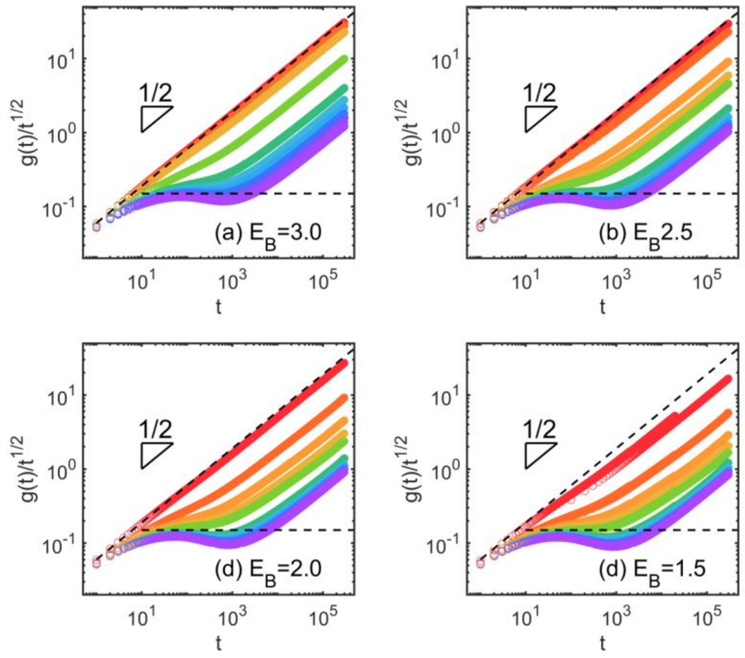

Figure S8. (a) to (d) are the results of mean-squared displacement of systems $E_B = 3.0$ to $E_B = 1.5$, respectively.

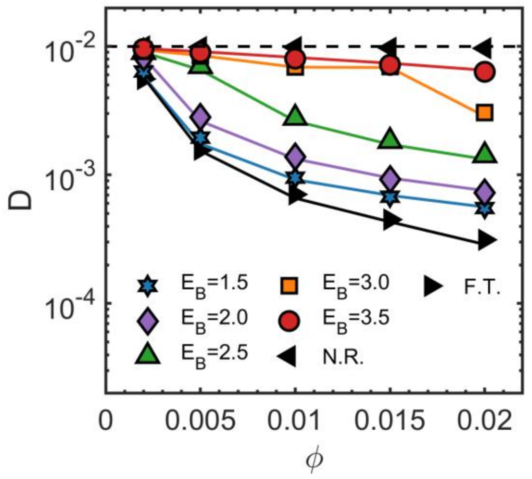

Figure S9. The diffusion coefficients D of the low-ϕ systems. The symbols are simulation results. The dark solid line is the prediction by the classical Rouse model for the F.T. systems. The other solid lines are the predictions by the modified Rouse model.

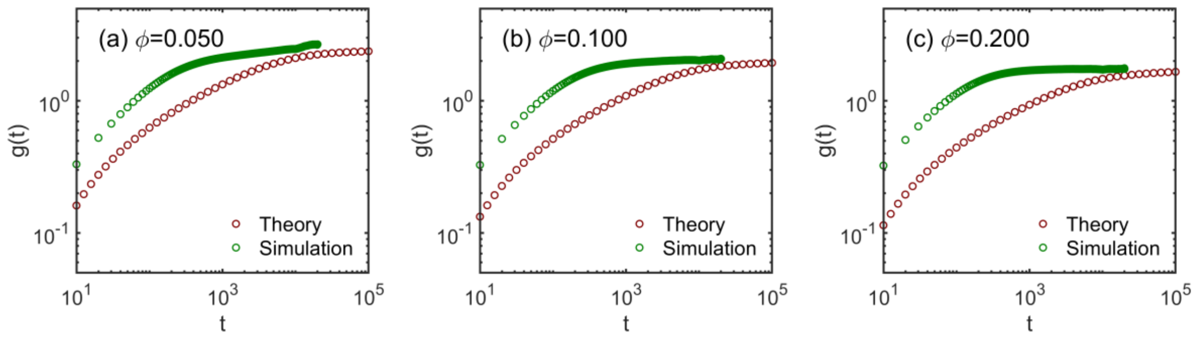

Figure S10. The results of mean-squared displacement of fixed-topology (F.T.) systems with $\phi \geq 0.05$. The MSD curves are capped as $t \rightarrow \infty$ because of the percolated networks and the permanent bonds. By assigning a cutoff distance and using the classical Rouse model, the theoretical prediction of the capped MSD values can be calculated. The predictions are compared with the simulation estimation of the F.T. systems. (a) to (c) are the results of systems with $\phi = 0.05$ to $\phi = 0.2$, respectively.

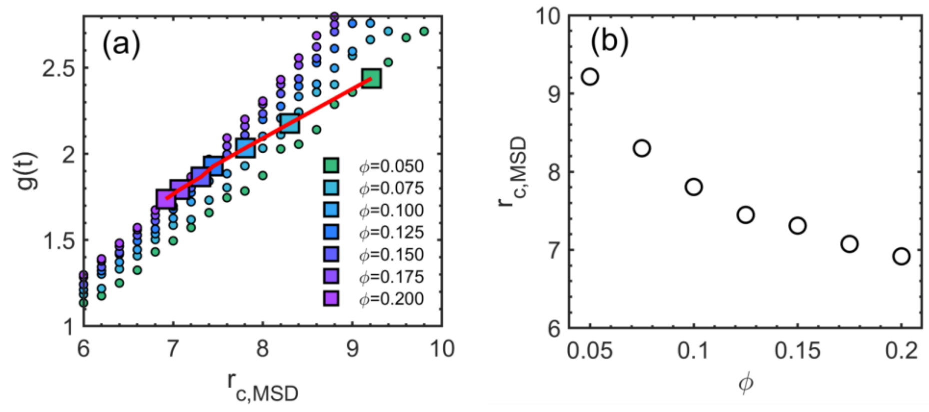

Figure S11. (a) By matching the theoretical prediciton of the capped MSD values and the simulation estimation of the F.T. systems, a correlation as shown in (b) between the volume fraction ϕ and the cutoff distance $r_{c,MSD}$ is established for the percolated systems.

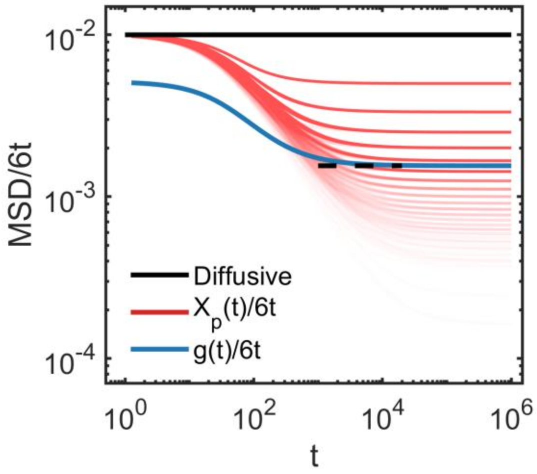

Figure S12. Relaxation dynamics estimated by the classical Rouse model. The contributions of individual modes to the bulk diffusion coefficient marked by the black dashed line. The results of the system with $E_B = 3.5$ and $\phi = 0.02$ are used as an example.

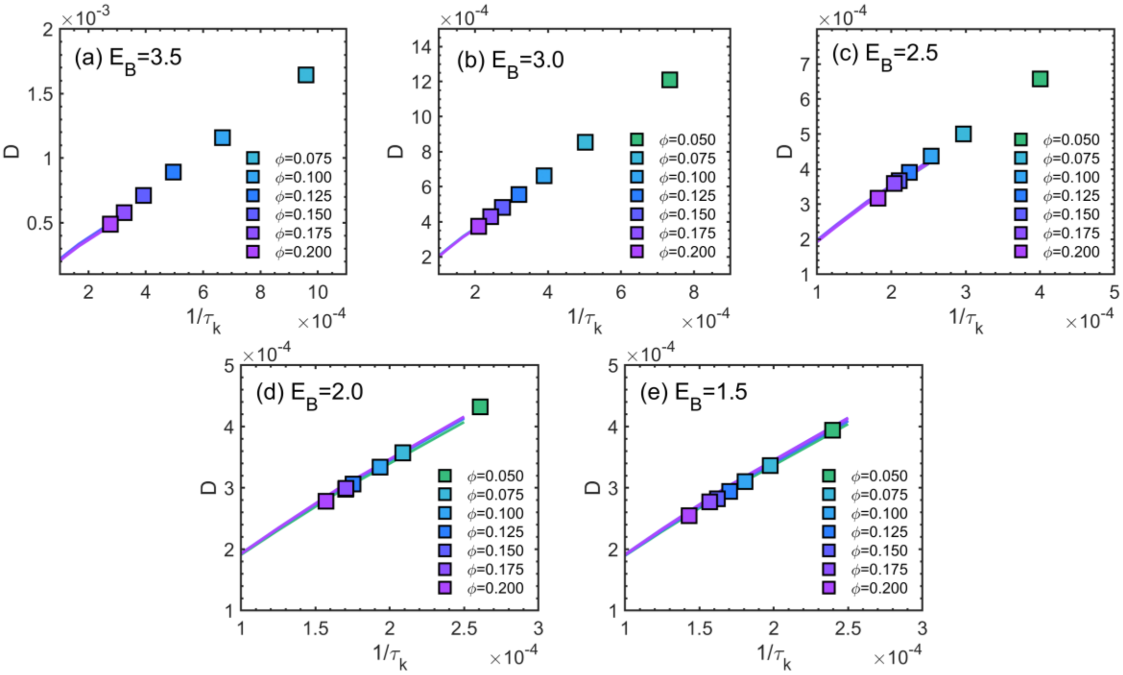

Figure S13. Diffusion coefficients estimated by the modified Rouse model. By assigning different τ_k values to the modified Rouse model, the diffusion coefficient D varies. By matching the theoretical predictions and the simulation results, the effective τ_k values are estimated. (a) to (e) are the results of systems with $E_B = 3.5$ to $E_B = 1.5$, respectively.

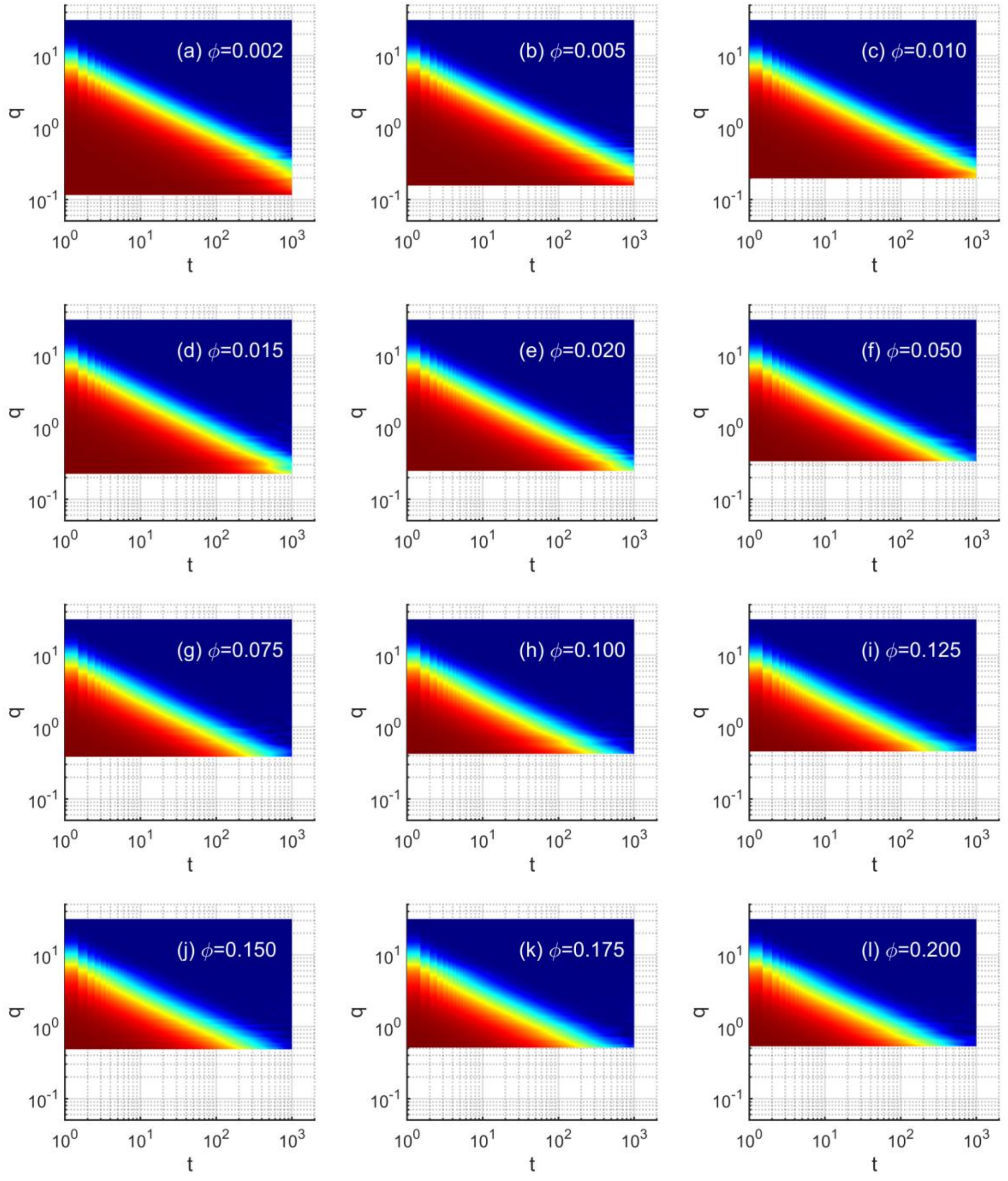

Figure S14. The dynamic structure factor of the no-reaction (N.R.) systems. The behaviors are simple liquid like. The dynamics structure factor decays exponetially and is insensitive to ϕ.

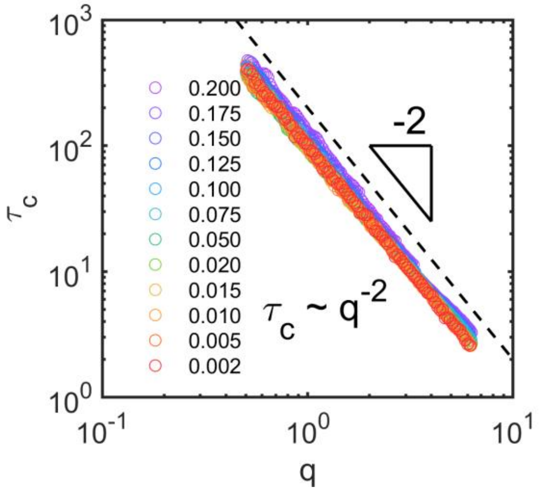

Figure S15. The characteristic relaxation times τ_c of the N.R. systems. The $\tau_c{\sim}q^{-2}$ scaling indicates a simple liquid-like behavior.

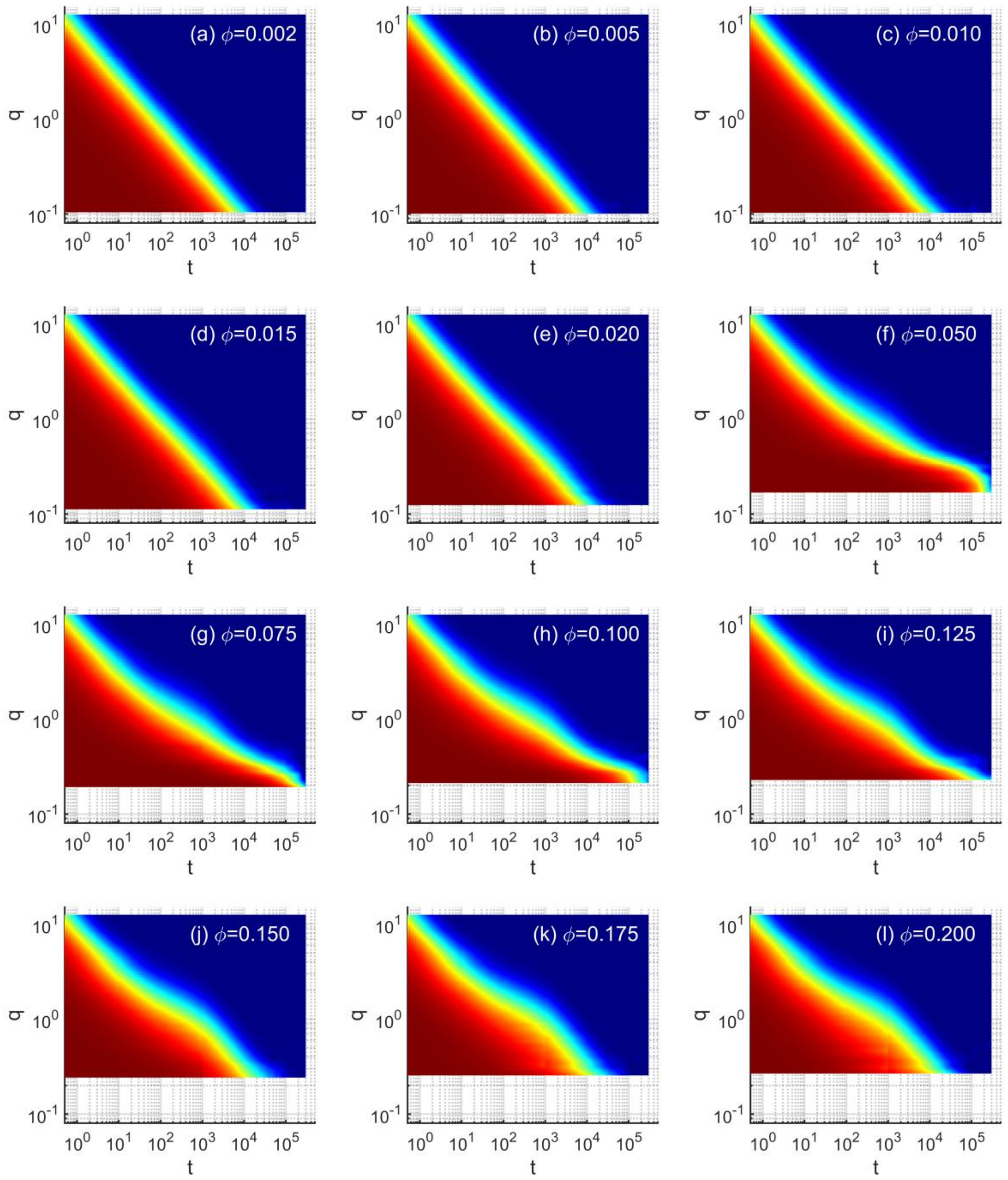

Figure S16. The dynamic structure factor of the systems with $E_B = 3.5$.

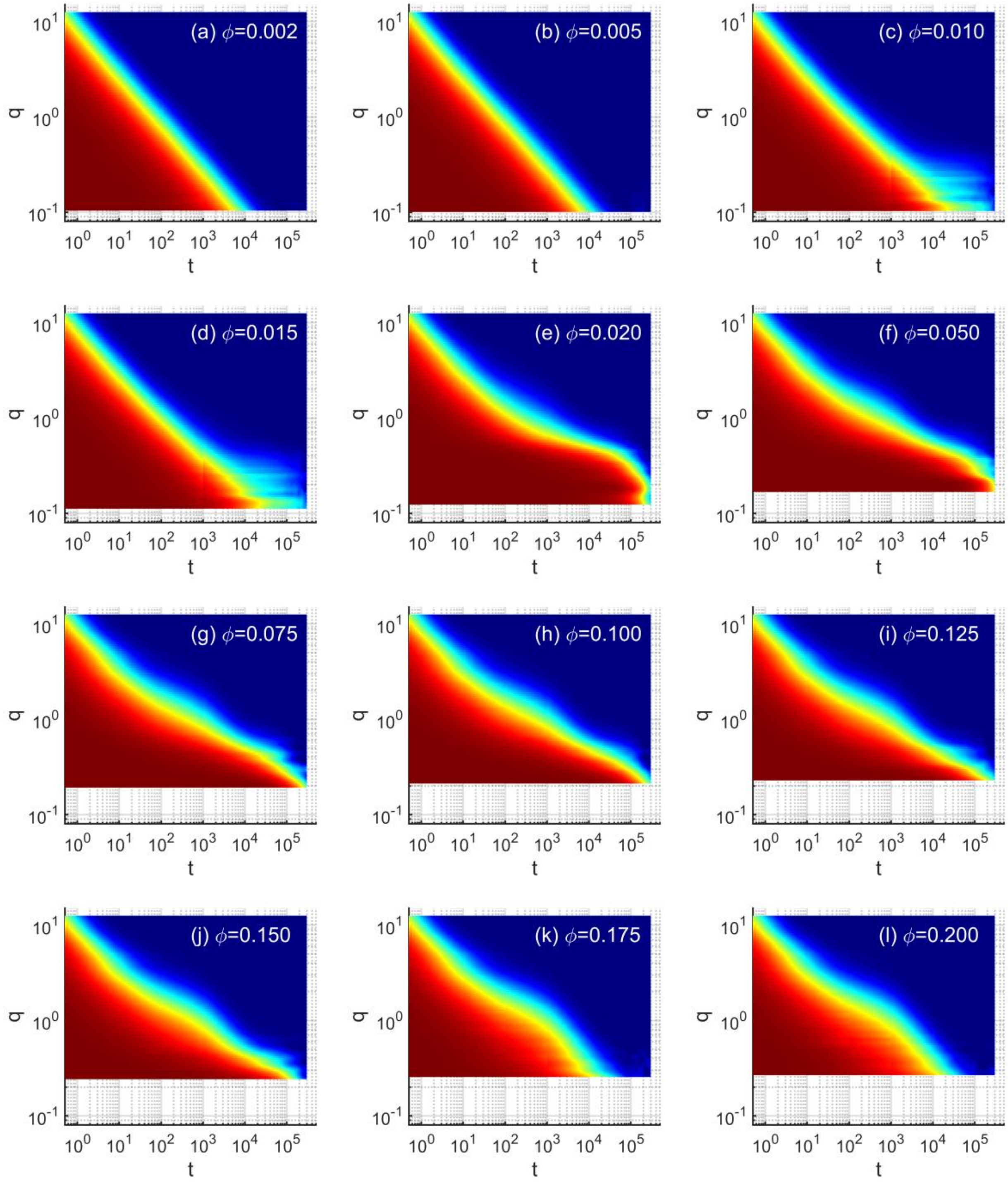

Figure S17. The dynamic structure factor of the systems with $E_B = 3.0$.

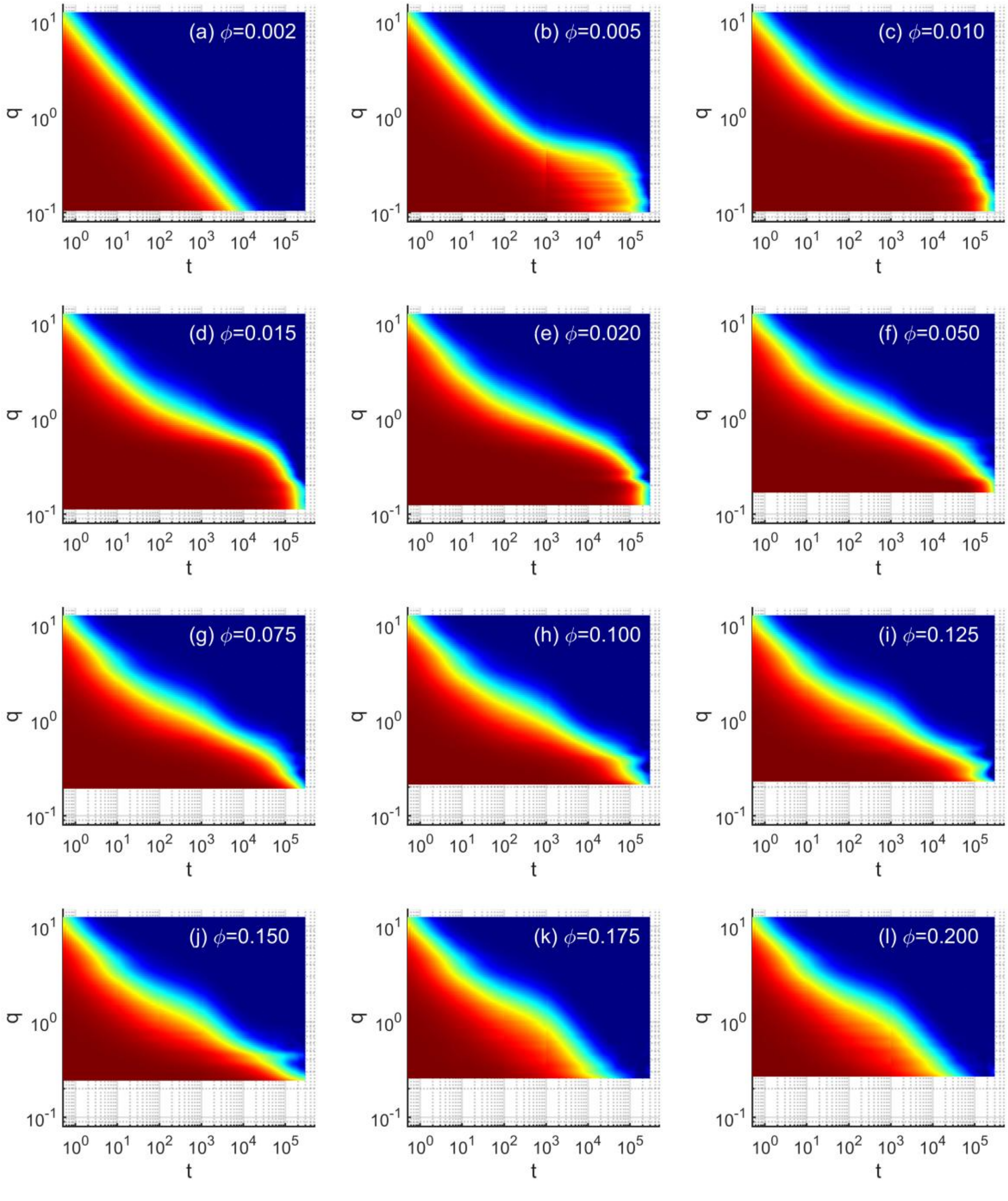

Figure S18. The dynamic structure factor of the systems with $E_B = 2.5$.

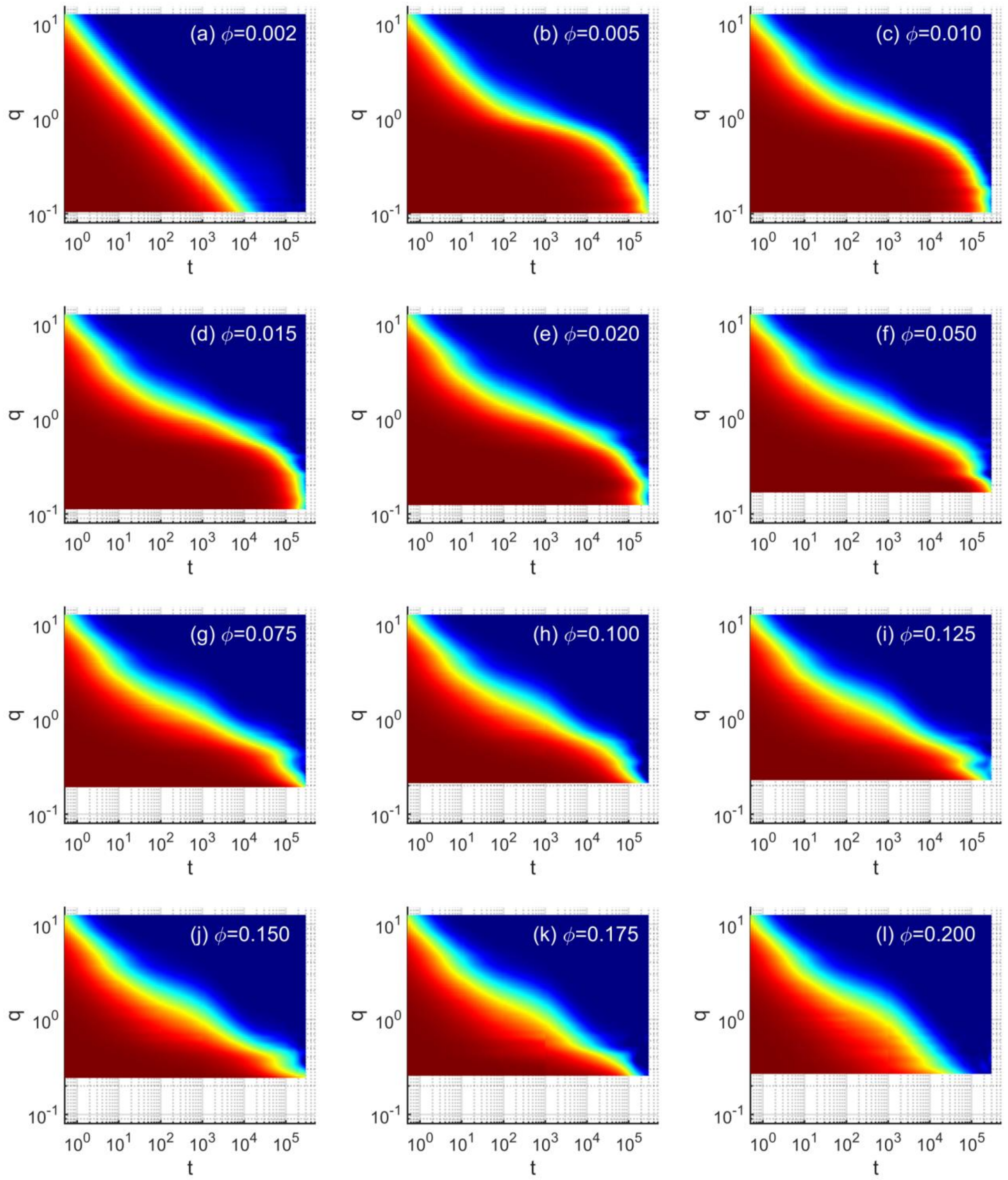

Figure S19. The dynamic structure factor of the systems with $E_B = 2.0$.

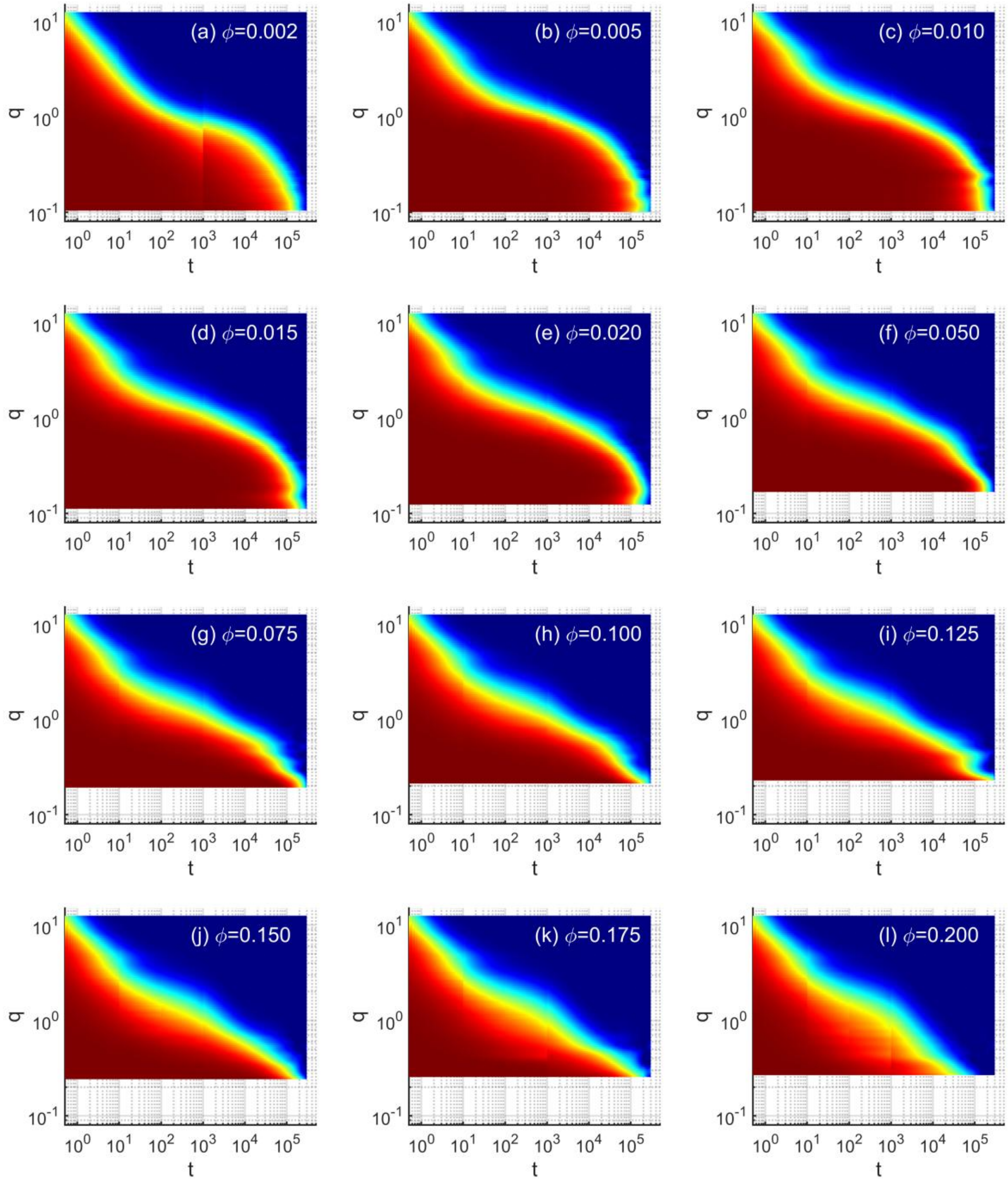

Figure S20. The dynamic structure factor of the systems with $E_B = 1.5$.

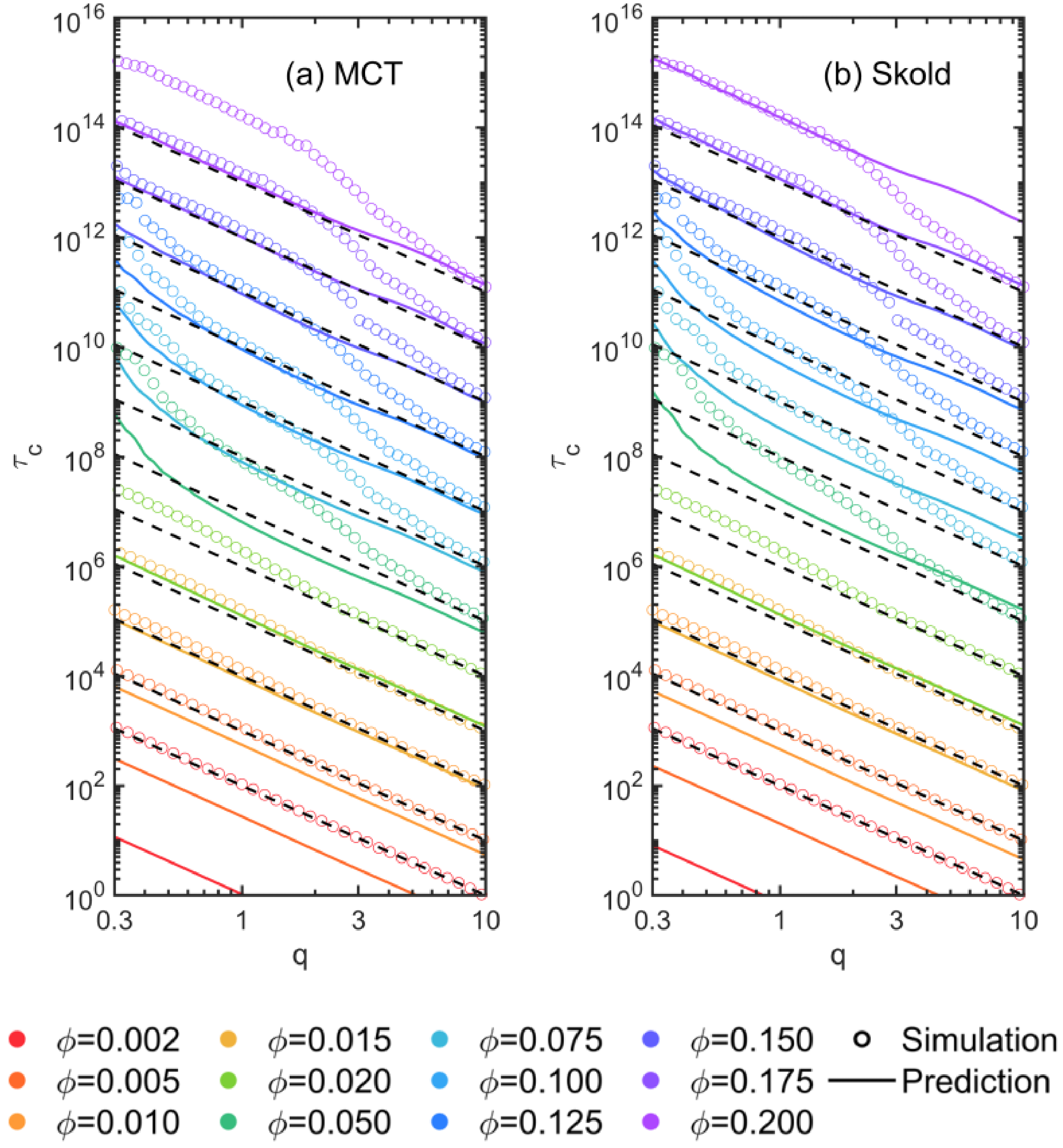

Figure S21. The characteristic time τ_c of the systems with $E_B = 3.5$.

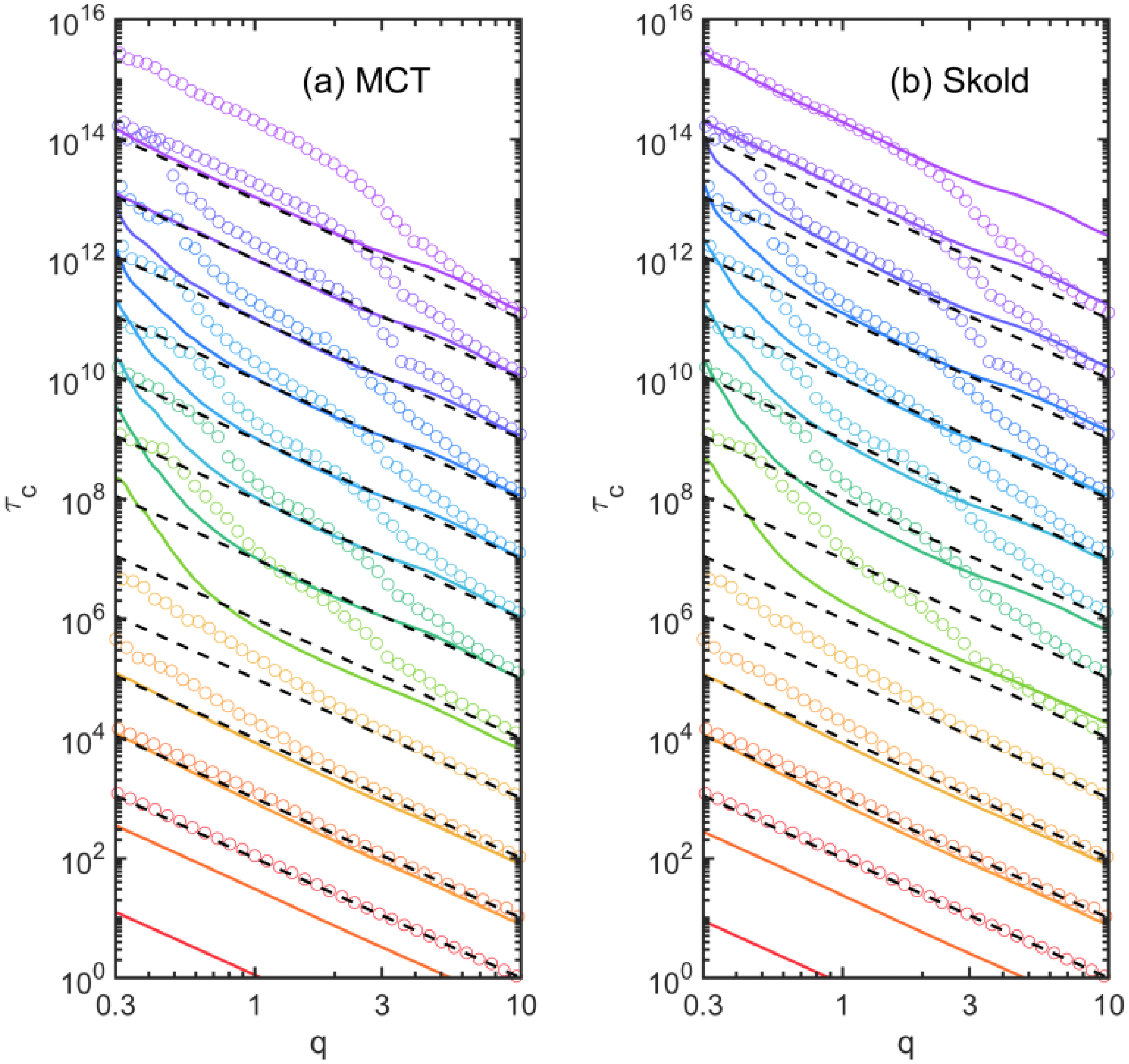

Figure S22. The characteristic time τ_c of the systems with $E_B = 3.0$.

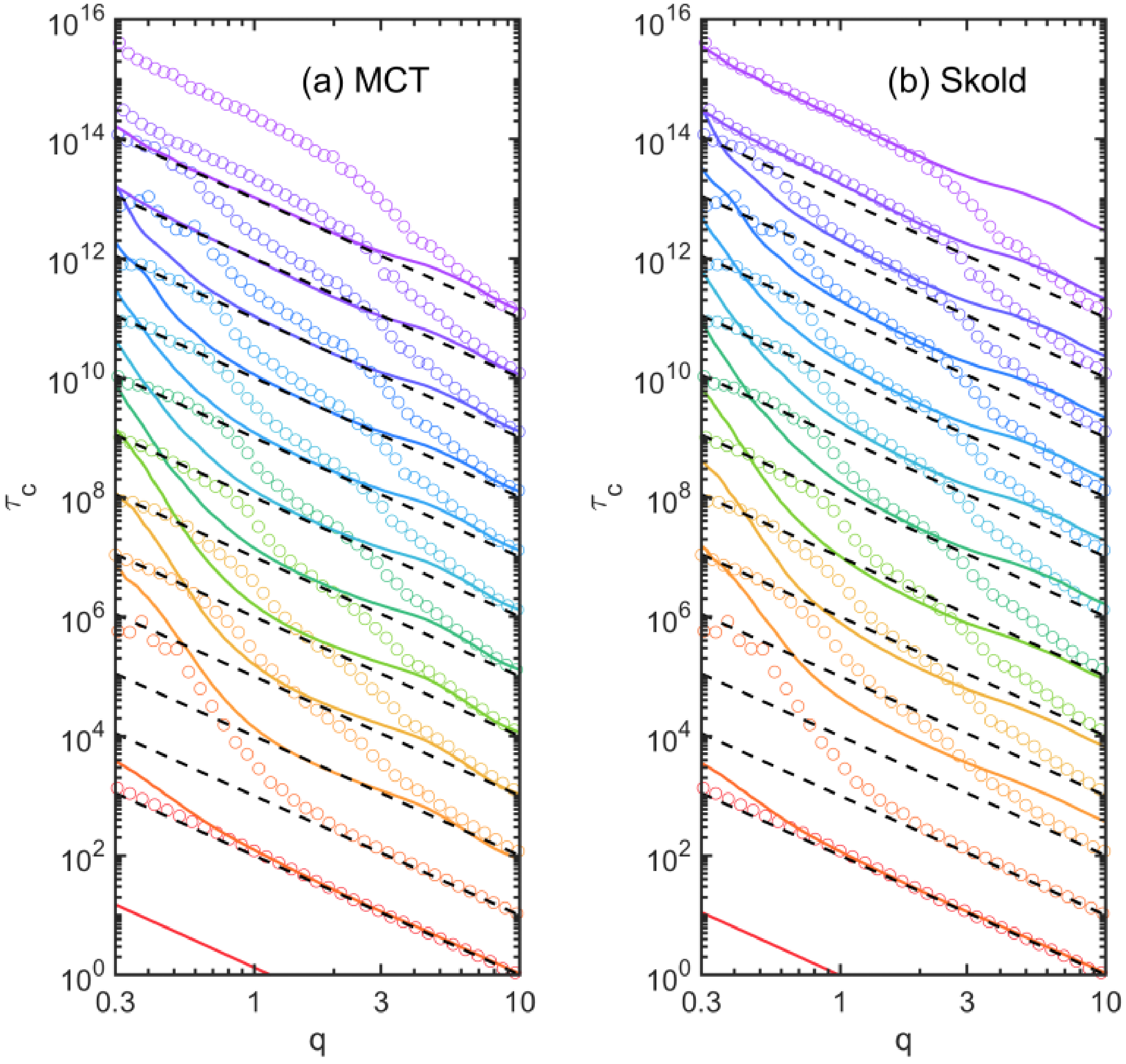

Figure S23. The characteristic time τ_c of the systems with $E_B = 2.5$.

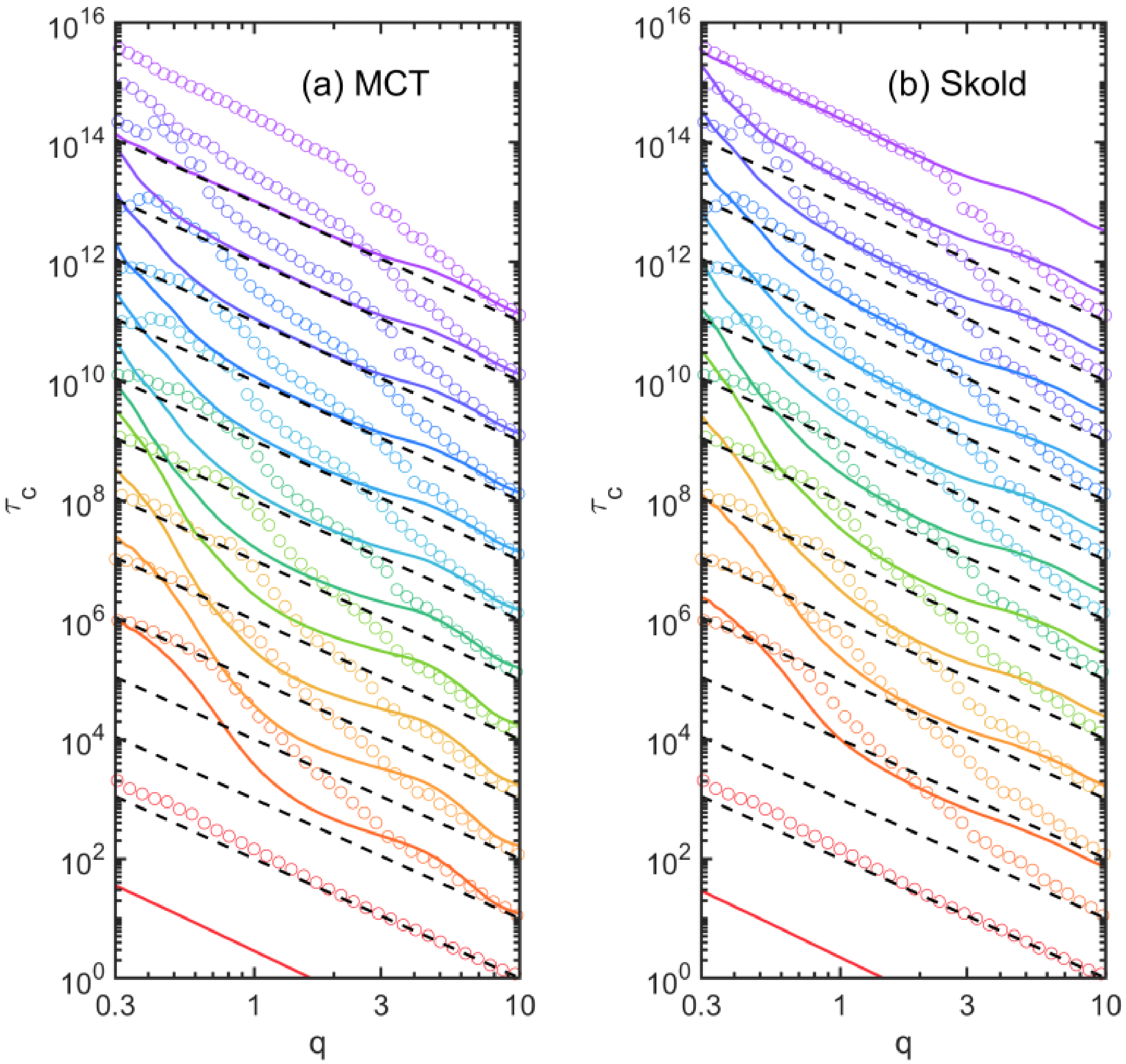

Figure S24. The characteristic time τ_c of the systems with $E_B = 2.0$.

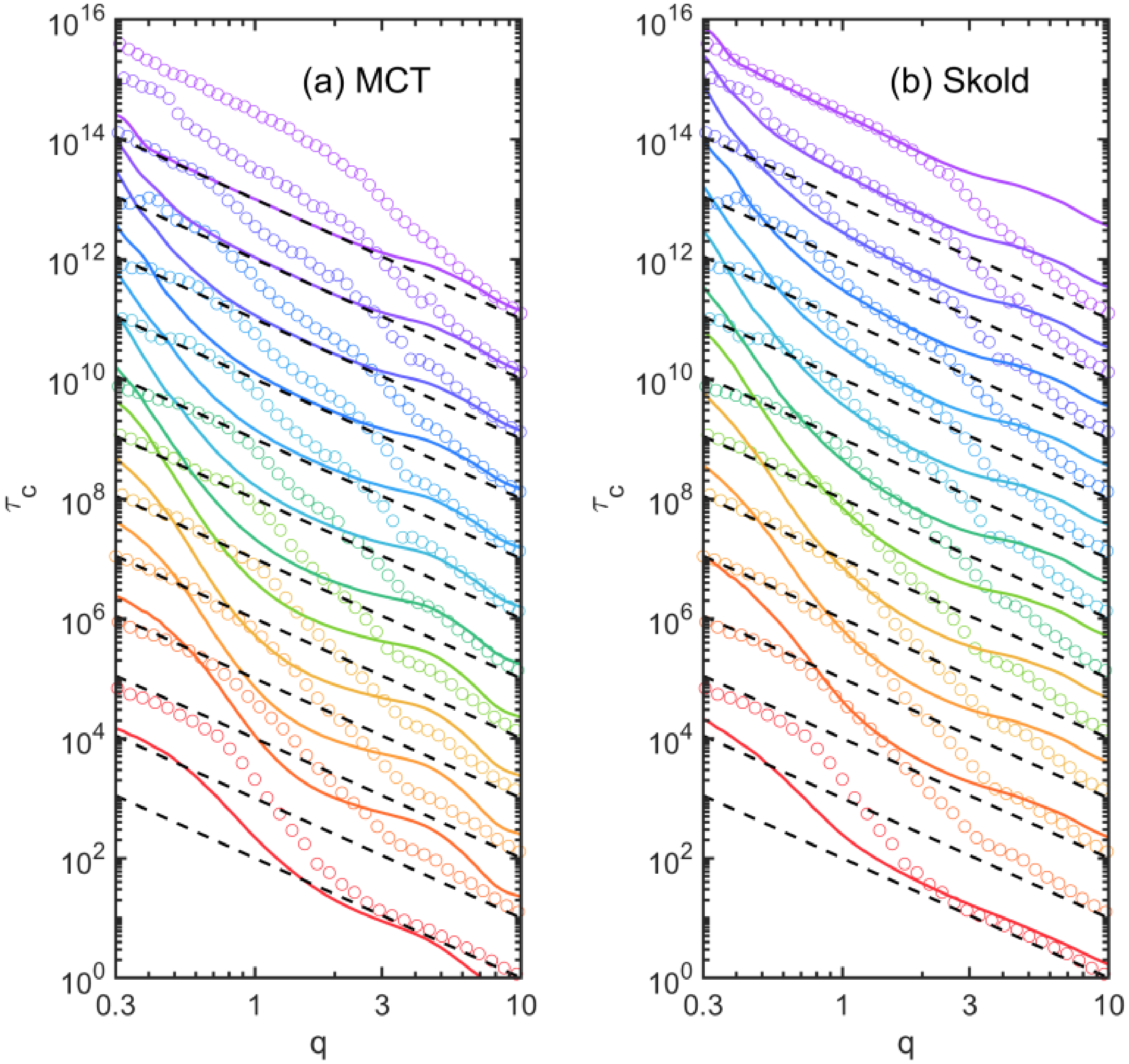

Figure S25. The characteristic time τ_c of the systems with $E_B = 1.5$.

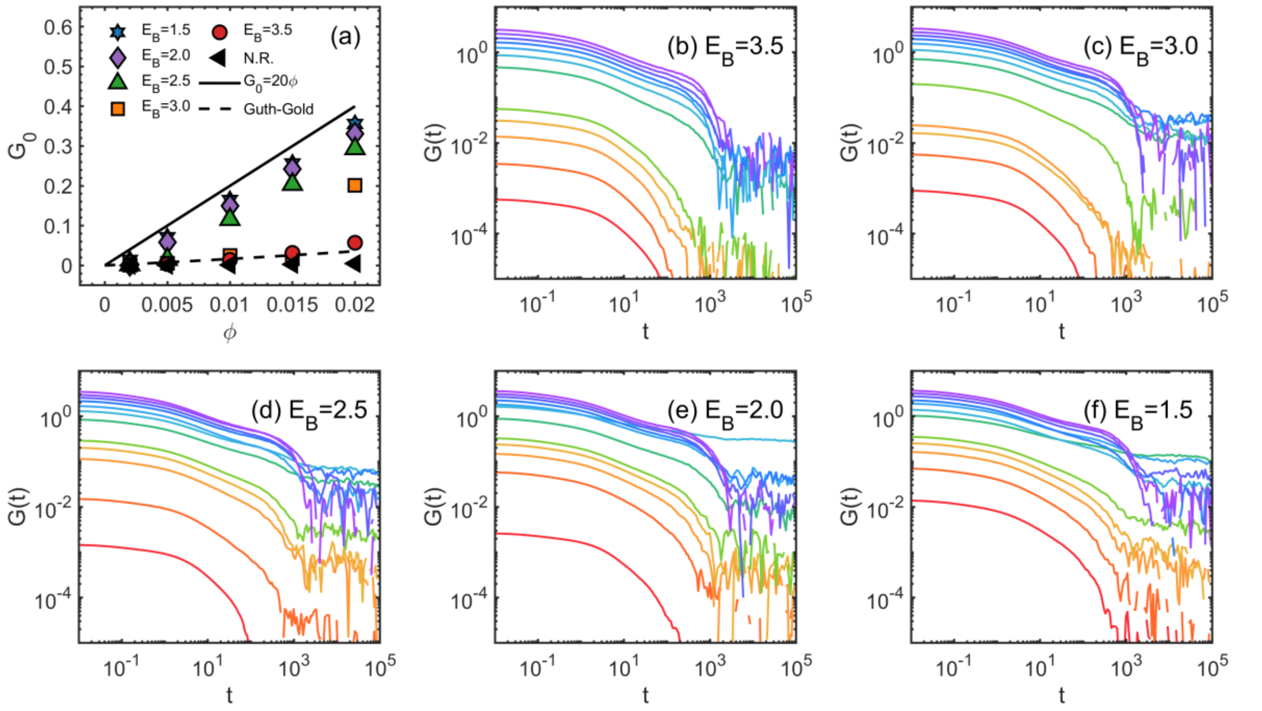

Figure S26. Relaxation modulus. (a) The magnitude of shear modulus G_0 as functions of E_B and ϕ. This is an zoom-in view of **Figure 10**a of the main text. While the results of the N.R. systems follow the Guth-Gold prediction, the model systems show a much stronger nonlinear reinforcement effect. (b) to (f) are the $G(t)$ results for the systems with $E_B = 3.5$ to $E_B = 1.5$, respectively.

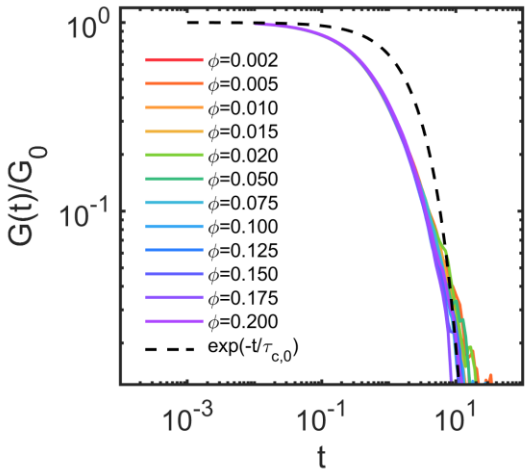

Figure S27. The relaxation modulus of the N.R. systems. All the simulation results collapse into a master curve that follow the exponetial decay with a characteristic time $\tau_{c,0}$.

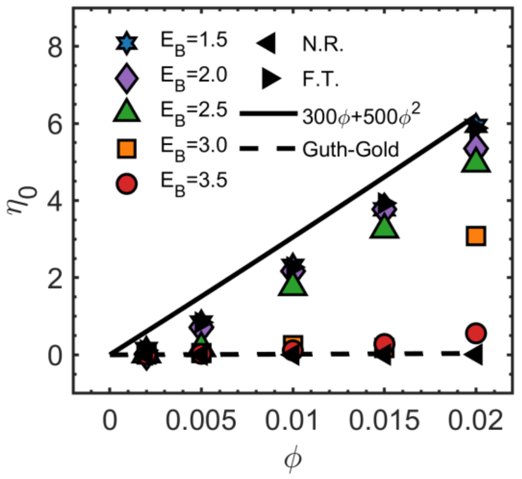

Figure S28. Viscosity η_0. This is a zoomed-in view of **Figure 11** in the main text.

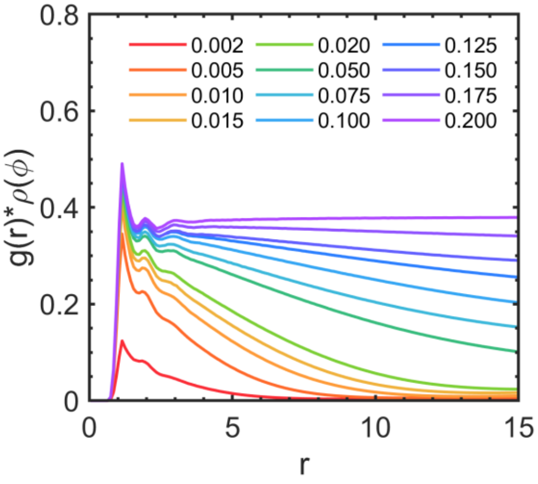

Figure S29. The rescaled radial distribution functions of the systems with $E_B = 1.5$.

References

(1) Rouse Jr, P. E. A Theory of the Linear Viscoelastic Properties of Dilute Solutions of Coiling Polymers. *J. Chem. Phys.* **1953**, *21* (7), 1272–1280.

(2) Lowen, H.; Szamel, G. Long-Time Self-Diffusion Coefficient in Colloidal Suspensions: Theory versus Simulation. *Journal of Physics: Condensed Matter* **1993**, *5* (15), 2295.

(3) Sköld, K. Small Energy Transfer Scattering of Cold Neutrons from Liquid Argon. *Phys. Rev. Lett.* **1967**, *19* (18), 1023.
